# Structure-Function Analysis of DipA, a *Francisella tularensis* Virulence Factor Required for Intracellular Replication

**DOI:** 10.1371/journal.pone.0067965

**Published:** 2013-06-26

**Authors:** Audrey Chong, Robert Child, Tara D. Wehrly, Dedeke Rockx-Brouwer, Aiping Qin, Barbara J. Mann, Jean Celli

**Affiliations:** 1 Laboratory of Intracellular Parasites, Rocky Mountain Laboratories, National Institute of Allergy and Infectious Diseases, National Institutes of Health, Hamilton, Montana, United States of America; 2 Department of Medicine, University of Virginia, Charlottesville, Virginia, United States of America; 3 Department of Microbiology, University of Virginia, Charlottesville, Virginia, United States of America; University of Rochester, United States of America

## Abstract

*Francisella tularensis* is a highly infectious bacterium whose virulence relies on its ability to rapidly reach the macrophage cytosol and extensively replicate in this compartment. We previously identified a novel *Francisella* virulence factor, DipA (FTT0369c), which is required for intramacrophage proliferation and survival, and virulence in mice. DipA is a 353 amino acid protein with a Sec-dependent signal peptide, four Sel1-like repeats (SLR), and a C-terminal coiled-coil (CC) domain. Here, we determined through biochemical and localization studies that DipA is a membrane-associated protein exposed on the surface of the prototypical *F. tularensis* subsp. *tularensis* strain SchuS4 during macrophage infection. Deletion and substitution mutagenesis showed that the CC domain, but not the SLR motifs, of DipA is required for surface exposure on SchuS4. Complementation of the *dipA* mutant with either DipA CC or SLR domain mutants did not restore intracellular growth of *Francisella* , indicating that proper localization and the SLR domains are required for DipA function. Co-immunoprecipitation studies revealed interactions with the *Francisella* outer membrane protein FopA, suggesting that DipA is part of a membrane-associated complex. Altogether, our findings indicate that DipA is positioned at the host–pathogen interface to influence the intracellular fate of this pathogen.

## Introduction

The Gram-negative intracellular bacterium Francisella tularensis is the causative agent of tularemia, a potentially fatal zoonosis affecting a variety of mammals, including humans [1]. Human tularemia can be contracted through multiple routes with the most acute form of disease resulting from inhalation of as few as 10 organisms [2]. Of the three subspecies of *F. tularensis*, subsp. *tularensis, holarctica*, and *mediasiatica*, the former two are responsible for the majority of human infections and disease [2]. In addition, *F* *. novicida* , a closely related species, is considered non-pathogenic for immunocompetent humans, yet retains high virulence in mice and is widely used as a model organism [3].

Given its pathogenic potential and renewed concerns for its misuse as a bioweapon, much research has focused on understanding the mechanisms of virulence of *F. tularensis*. Although *F. tularensis* can infect a range of host cells that include hepatocytes, neutrophils, fibroblasts and endothelial cells, a key virulence trait is the ability of *F. tularensis* to reside within mononuclear phagocytes [4–7]. In particular, macrophages are considered an important target for infection within which *F. tularensis* demonstrates a multifaceted lifecycle that is essential to its pathogenesis [4,8]. Upon internalization, the bacterium transiently resides within a phagosome from which it rapidly escapes to reach the macrophage cytosol [9–15]. Cytosolic bacteria undergo extensive replication, induce macrophage apoptosis or pyroptosis, and eventual egress from infected cells [16–19]. A subset of post-replicative bacteria is re-captured into endocytic vacuoles in murine bone marrow-derived macrophages (BMMs) through an autophagy related process [11]. A number of *Francisella* factors that contribute to various aspects of its complex intracellular lifecycle have been identified (reviewed in 20,21). The most prominent virulence determinant is the *Francisella* pathogenicity island (FPI), a 30-kb locus that encodes components of a type VI secretion system (T6SS) [22,23]. Several non-FPI encoded factors have also been shown to contribute to *Francisella* pathogenesis, although many of these, as well as those encoded by genes within the FPI, show no homology to known bacterial proteins and thus, their specific functions remain unclear [10,24–31].

Previously, we identified a *Francisella* -specific locus that was transcriptionally upregulated in *F. tularensis* subsp. *tularensis* strain SchuS4 during the cytosolic replication stage of BMM infection [10]. Deletion of *dipA* in SchuS4 did not affect phagosomal escape, but impaired intracellular replication and survival in BMMs. Furthermore, the SchuS4Δ*dipA* mutant was defective for replication, dissemination and lethality in mice, demonstrating that *dipA* encodes a *bona fide* virulence factor [10]. The *dipA* locus encodes a novel protein predicted to contain several conserved domains, four Sel1-like repeats (SLRs) and a coiled-coil (CC) motif, that are implicated in protein–protein interactions. Aside from these domains, DipA has little similarity to other known proteins and is conserved among the subspecies, suggesting a potentially unique, *Francisella* -specific, molecular mechanism of virulence.

In the present study, we examined the biochemical and structural characteristics of DipA to gain insight into its role in *F. tularensis* pathogenesis. We report that DipA is a membrane-associated protein localized on the bacterial surface during macrophage infection, and show that the SLR and CC domains are functionally distinct. We also identified a *Francisella* outer membrane protein, FopA, that interacts with DipA, suggesting that DipA may be part of a membrane complex.

## Results and Discussion

### DipA is localized to the surface of *F. tularensis* subsp. *tularensis* SchuS4

**Figure 1:**
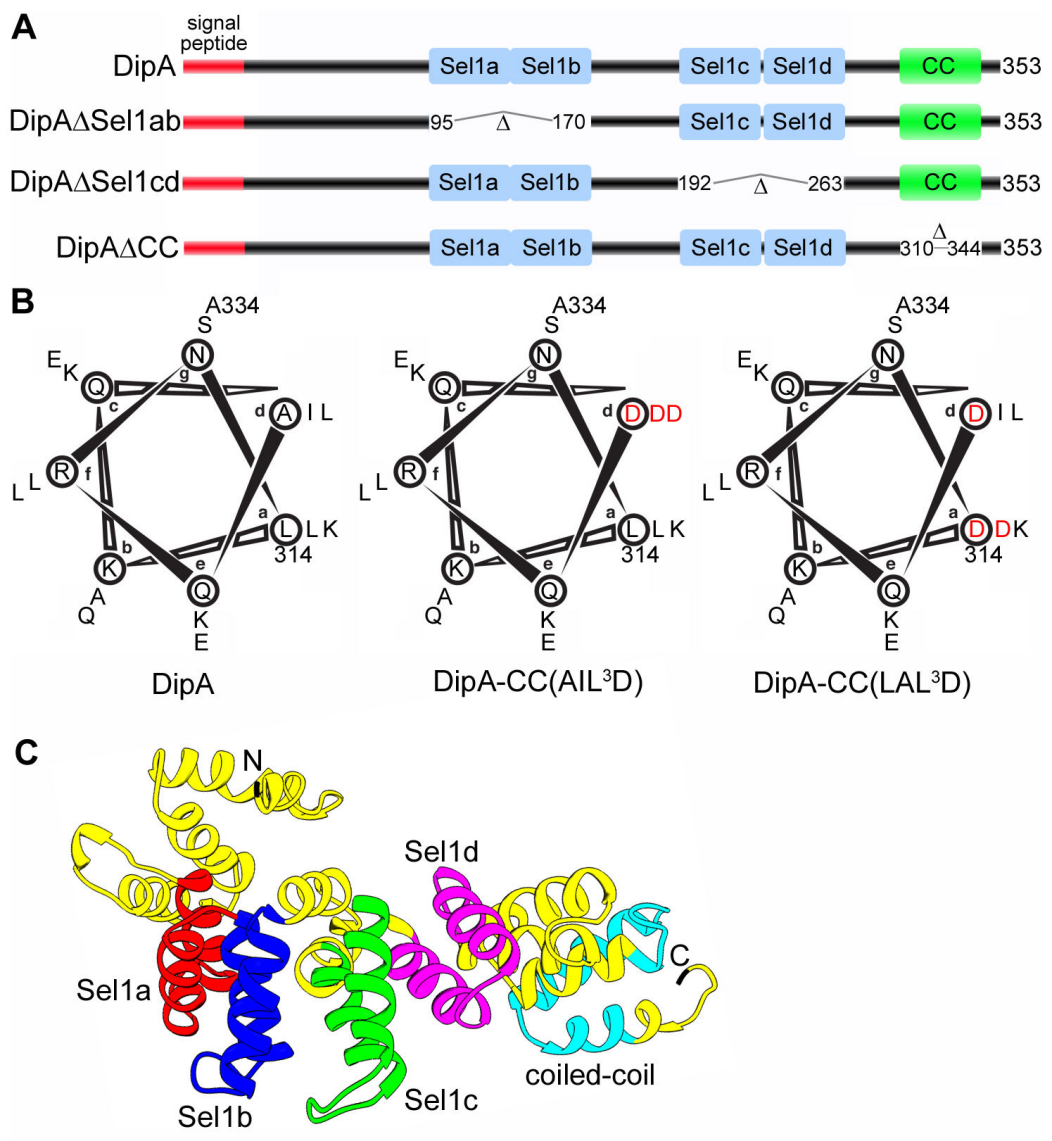
Predicted structure of DipA. (A) Schematic representations of DipA and domain deletion mutants generated in this study. The predicted N-terminal 20 amino acid signal peptide is denoted is red, the Sel1-like repeat domains are denoted in blue and the coiled-coil (CC) domain is denoted in green. Domain deletion mutants were designed to encompass deletion of Sel1a and Sel1b domains (DipAΔSel1ab), Sel1c and Sel1d domains (DipAΔSel1cd), and the CC domain (DipAΔCC). (B) Helical wheel representations of the CC domain corresponding to amino acid residues of DipA. Heptad-repeat positions are denoted *a* to *g*. Two DipA CC domain substitution mutants where three hydrophobic residues in core positions *a* and *d* were mutated to aspartate [DipACC(AIL ^3^D) and DipACC(LAL ^3^D)]. Mutations are indicated in red. (C) Three-dimensional ribbon model of DipA predicted by I-TASSER and visualized using Chimera software. The Sel1a domain corresponding to residues 96-132 is indicated in red, the Sel1b domain corresponding to residues 133-169 is indicated in blue, the Sel1c domain corresponding to residues 193-229 is indicated in green, the Sel1d domain corresponding to residues 231-262 is indicated in purple, and the CC domain corresponding to residues 311-343 is indicated in cyan.

**Figure 2:**
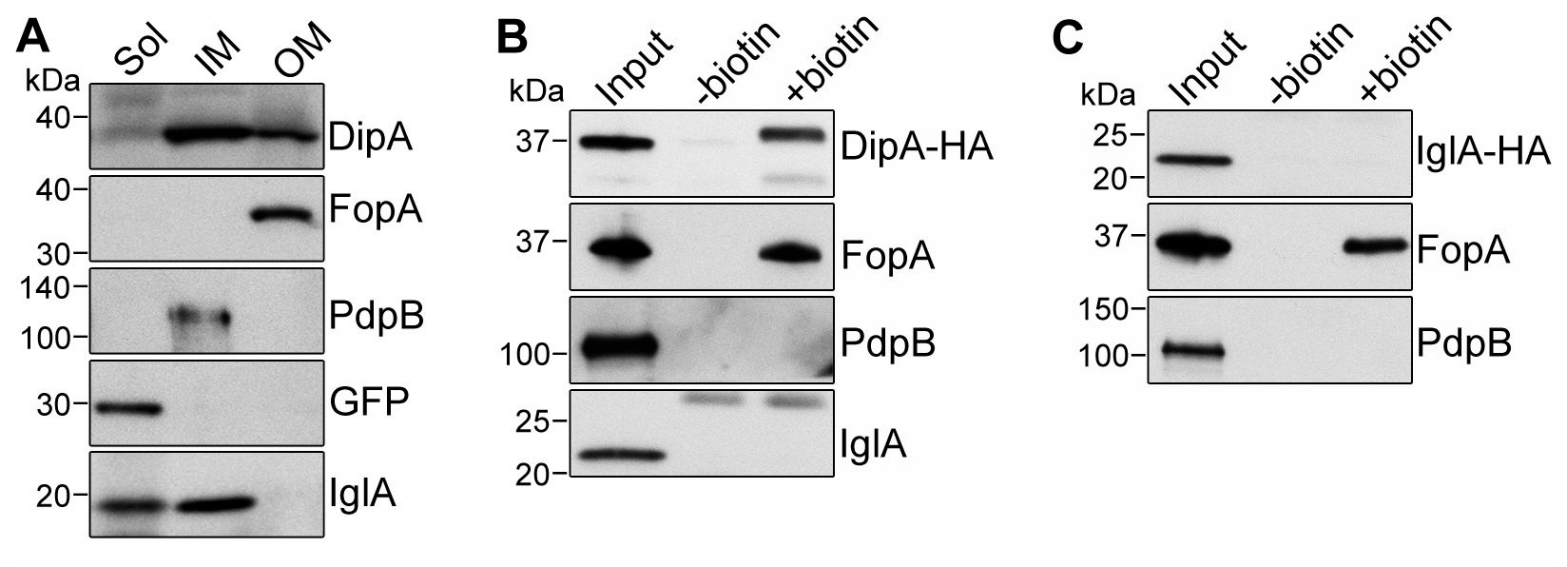
DipA is a surface-exposed, membrane-associated protein. (A) Subcellular localization of DipA, FopA, PdpB, GFP, and IglA from GFP-expressing SchuS4. Soluble (Sol), inner membrane (IM), and outer membrane (OM) enriched fractions were separated based on Sarkosyl solubility and subjected to immunoblot analysis with antibodies against DipA, FopA, PdpB, GFP and IglA. Each fraction was concentrated to the same volume and equal volumes were loaded. GFP, PdpB and FopA were used as soluble, inner membrane and outer membrane markers, respectively. (B and C) Immunoblot analysis of purified surface biotinylated proteins from SchuS4Δ*dipA*(p*dipA-HA*) (B) or SchuS4(p*iglA-HA*) (C) lysates. DipA-HA and IglA-HA were detected using anti-HA antibodies. FopA was used as a positive control; PdpB and IglA were used as negative controls. Input, untreated (-biotin) and biotinylated (+biotin) samples were processed for CFU enumeration and immunoblotting as described in Materials and Methods. Samples were loaded based on CFU equivalents as follows: 1x10^7^ (Input) or 1x10^8^ (-/+ biotin) for anti-DipA-HA analysis, 5x10^6^ (Input) or 1x10^8^ (-/+ biotin) for anti-FopA analysis, 1x10^7^ (Input) or 5x10^8^ (-/+ biotin) for anti-PdpB analysis, 1x10^7^ (Input) or 5x10^8^ (-/+ biotin) for anti-IglA analysis, 1x10^7^ (Input) or 5x10^8^ (-/+ biotin) for anti-IglA-HA analysis.

To characterize the functional role of DipA, we first analyzed the translated amino acid sequence for conserved domains using protein structure prediction programs (SMART, Pfam, COILS, and Marcoil). DipA is a 353 amino acid protein predicted to possess a 20 amino acid Sec-dependent signal peptide, four Sel1-like repeat (SLR) domains, and a C-terminal coiled-coil (CC) domain (Figure 1A and 1C). The signal peptide suggests that DipA may be secreted in a Sec-dependent manner, while the SLR and CC domains are ubiquitous structural motifs known to facilitate protein–protein interactions. Immunoblot analysis of bacterial subcellular fractions revealed that DipA, although detected in all fractions, was predominantly localized to the inner and outer membrane enriched fractions of GFP-expressing SchuS4 (SchuS4 GFP) (Figure 2A), indicating an extracytoplasmic location as predicted by the signal peptide. Known outer membrane (FopA), inner membrane (PdpB), and soluble (GFP) proteins were detected only in the expected fractions, indicating effective fraction separation (Figure 2A) [28,32–36]. In addition, we examined the localization of IglA in SchuS4 GFP because IglA was included as a control throughout this study. In *F* *. novicida* , IglA has been detected in the cytoplasm, outer membrane and membrane insoluble fractions, and the ambiguity of its distribution has been ascribed to its association with a macromolecular structure spanning the periplasm from the inner membrane [36–38]. Consistent with this model, we detected IglA in the soluble and inner membrane fractions of SchuS4 GFP (Figure 2A).

The presence of DipA in the bacterial outer membrane prompted us to ascertain whether DipA is localized to the bacterial surface. We used a SchuS4Δ*dipA* strain expressing DipA with a C-terminal tandem HA tag (DipA-HA) because of the higher sensitivity of the monoclonal anti-HA antibody. In surface protein biotinylation experiments, DipA-HA was detected in the biotinylated fraction (Figure 2B), revealing its exposure on the bacterial surface. FopA and PdpB served as positive and negative controls, respectively. Consistent with previous findings, FopA, but not PdpB, showed exposure on the bacterial surface (Figure 2B) [38,39]. IglA was previously detected on the surface of LVS and *F* *. novicida* strains over-expressing PdpD [38,39]. Contrary to these reports, but consistent with the fractionation properties reported here (Figure 2A) and by de Bruin et al. [37], IglA was not detected on the bacterial surface (Figure 2B). Surface biotinylation of IglA was also not detected in SchuS4 overexpressing IglA fused with an HA epitope arguing against the possibility of detection sensitivity of our methods, and ensuring that biotinylation of DipA-HA reflects true surface exposure and not an artefact of over-expression (Figure 2C). Furthermore, none of the proteins examined were detected in the absence of biotin labelling, confirming the specificity of streptavidin binding to biotinylated surface proteins (Figure 2B and 2C).

**Figure 3:**
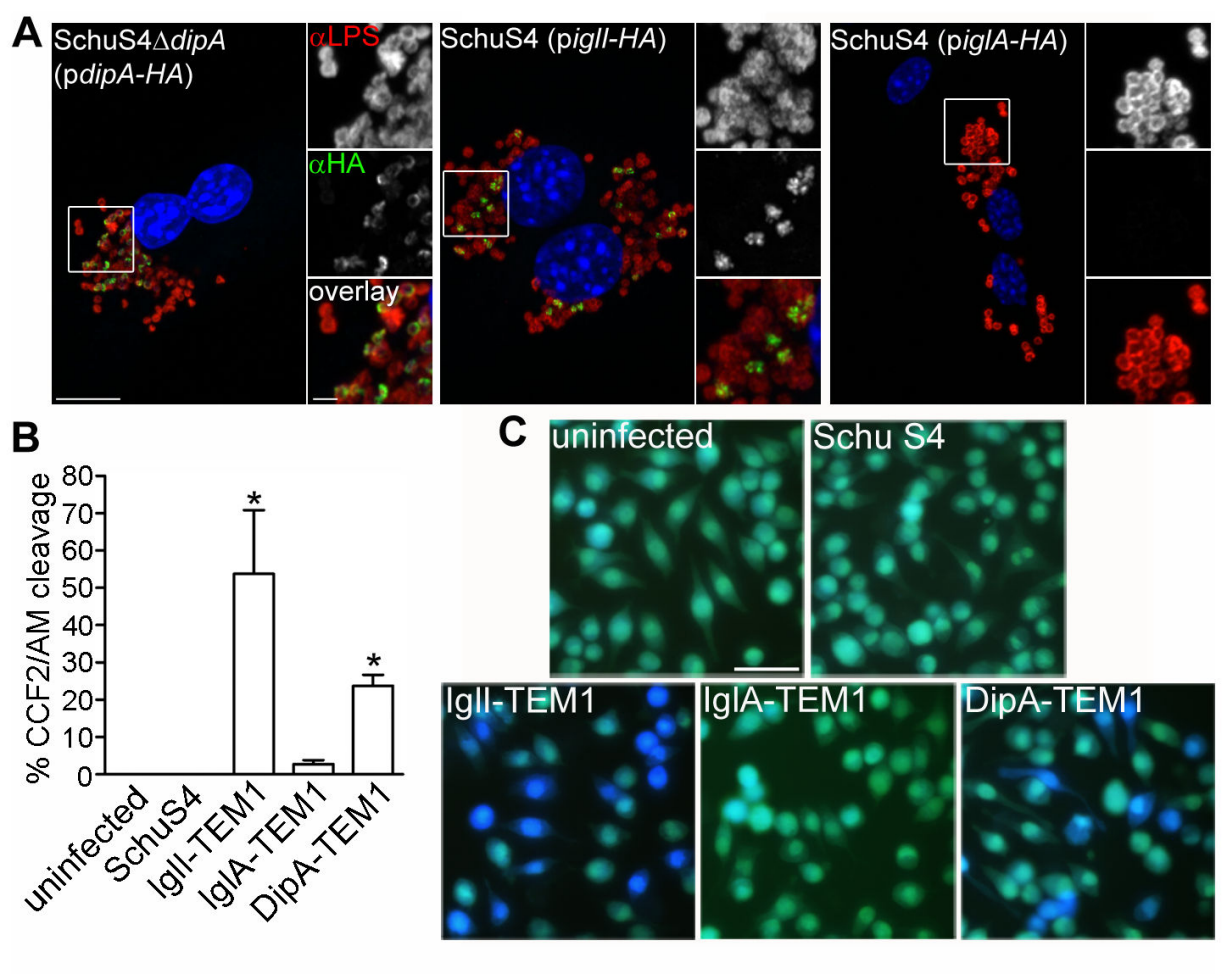
DipA is exposed to the host cytosol during macrophage infection. (A) Representative confocal micrographs of BMMs infected for 10 h with SchuS4Δ*dipA*(p*dipA-HA*), SchuS4(p*iglI-HA*), or SchuS4(p*iglA-HA*). Using conditions that permeabilize host plasma membranes but not bacterial membranes (described in Materials and Methods), samples were processed for immunofluorescence labelling of HA-tagged proteins (green) and bacterial LPS (red), and counterstained with DAPI to label DNA (blue). Magnified insets show single channel images of the boxed area. Scale bars, 10 or 2 µm. (B) Quantification of CCF2/AM cleavage in J774A.1 cells that were either uninfected or infected with SchuS4, or SchuS4 expressing C-terminal TEM1 fusions with IglI, IglA or DipA. After 16 h, infected macrophages were loaded with CCF2/AM and analyzed by live cell microscopy for blue fluorescence emission. At least 100 cells were scored per experiment. Data are means ± SD from a representative experiment performed in triplicate out of three independent repeats. Asterisks indicate statistically significant differences compared to uninfected, SchuS4-infected, and SchuS4 expressing IglA-TEM1-infected controls (* *P < 0.05*, 1-way ANOVA, Tukey’s post-test). (C) Representative fluorescence micrographs of J774A.1 cells that were either uninfected or infected for 16 h with SchuS4, SchuS4 expressing IglI-TEM1, SchuS4 expressing IglA-TEM1, and SchuS4 expressing DipA-TEM1. Cells emitting blue fluorescence indicate delivery of TEM1 β-lactamase fusions to the cytosol and CCF2/AM cleavage. Intact CCF2/AM, indicating the absence of TEM1 β-lactamase activity in the cytosol, results in green fluorescence emission. Scale bar, 50 µm.

Since DipA is surface-exposed on bacteria grown *in vitro*, we next examined its localization in the context of a macrophage infection. Under conditions that do not permeabilize bacterial membranes, we detected DipA-HA on the surface of intracellular bacteria at 10 h post infection (p.i.) in BMMs by immunofluorescence labelling (Figure 3A). IglI-HA was similarly detected, consistent with previous reports of IglI secretion by *F. tularensis* during macrophage infection [35,40]. Co-staining with anti-LPS antibodies revealed co-localization of both DipA-HA and IglI-HA with bacterial surface structures (Figure 3A). Both DipA-HA and IglI-HA were only detected on the surface of a subset of bacteria either within BMMs (Figure 3A) or grown *in vitro* (data not shown). Since this variable staining pattern was not confined to an infection setting or to DipA only, it suggests a detection issue rather than differential surface expression within the bacterial population. In contrast, IglA-HA was not detected on the surface SchuS4 during BMM infection, corroborating our biochemical observations from *in vitro* grown bacteria. Lack of IglA-HA immunodetection was not due to lack of expression since DipA-HA, IglI-HA, and IglA-HA showed nearly equivalent levels of expression (Figure S1A).

To verify these observations, we used a fluorescence-based assay that relies on the accessibility of TEM1 β-lactamase protein fusions to the host cytosol to cleave and disrupt the fluorescence resonance energy transfer (FRET) of the β-lactamase-sensitive fluorescent substrate CCF2/AM. Expression of the TEM1-fusion proteins in SchuS4 was confirmed by immunoblot analysis and revealed comparable levels among the constructs (Figure S1B). At 16 h p.i., none of the uninfected and SchuS4-infected J774A.1 macrophage-like cells displayed CCF2/AM cleavage via blue fluorescence emission, indicating a low background level of intrinsic β-lactamase activity (Figure 3B and 3C), despite the presence of the *blaA1* (FTT0681c) and *blaB1* (FTT0611c) genes in the SchuS4 genome [41]. In contrast, a significantly higher percentage of J774A.1 cells emitting blue fluorescence were detected upon infection with SchuS4 strains expressing IglI-TEM1 (53.8 ± 17.0%; Figure 3B and C), in agreement with previous findings demonstrating IglI secretion via the FPI-encoded T6SS [35,40,42]. Infection with a SchuS4 strain expressing DipA-TEM1 also resulted in blue fluorescence emission (23.8 ± 2.9% of J774A.1 cells), indicating delivery of DipA-TEM1 to the host cytosol (Figure 3B and 3C). As a negative control, very few macrophages displaying CCF2/AM conversion were detected when infected with SchuS4 expressing IglA-TEM1 (2.7 ± 1.1%; Figure 3B and 3C). Because *F. tularensis* is a cytosolic pathogen, this assay does not discriminate between DipA secretion into the macrophage cytosol or exposure to the cytosol due to its localization on the bacterial surface. Altogether, our results show that DipA is a membrane-associated protein exposed both on the surface of *in vitro* grown *Francisella* and to the cytosol of infected macrophages.

### The Sel1-like repeat domains are important for DipA function

**Figure 4:**
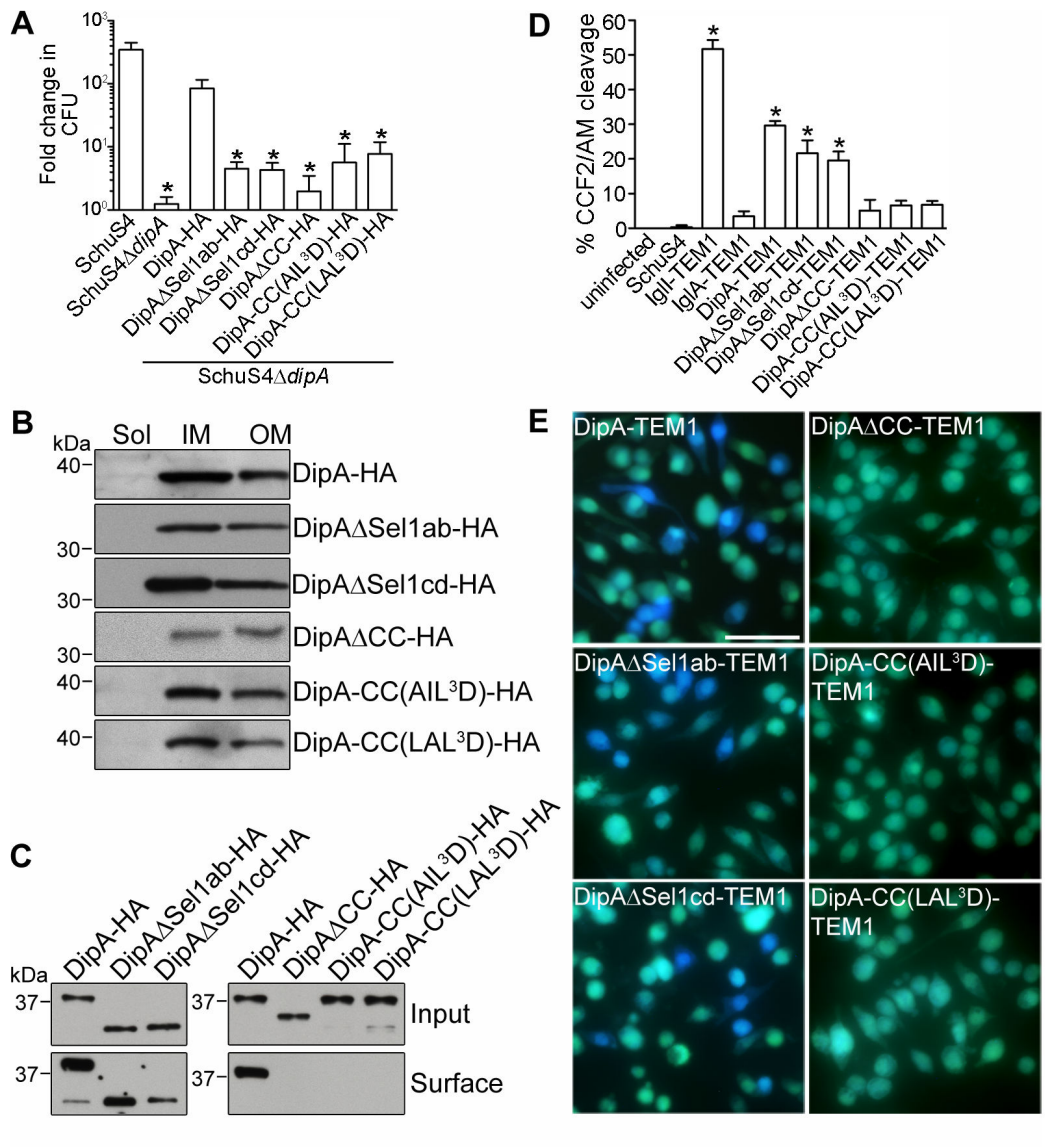
The SLR and CC domains of DipA are functionally distinct. (A) Ability of DipA variants to complement the intracellular growth defect of SchuS4Δ*dipA*. Viable intracellular bacteria were enumerated at 1 h and 16 h p.i. from BMMs infected with SchuS4, SchuS4Δ*dipA*, or SchuS4Δ*dipA* expressing HA-tagged DipA variants (DipA-HA, DipAΔSel1ab-HA, DipAΔSel1cd-HA, DipAΔCC-HA, DipACC(AIL ^3^D)-HA, or DipACC(LAL ^3^D)-HA). Fold change in replication was calculated by comparing CFUs at 16 h p.i. versus 1 h p.i. Data are means ± SD from three independent experiments. Asterisks indicate statistically significant differences compared to SchuS4-infected and SchuS4Δ*dipA* expressing DipA-HA-infected macrophages (* *P < 0.05*, 1-way ANOVA, Tukey’s post-test). (B) Subcellular localization of HA-tagged DipA variants as described in (A). Soluble (Sol), inner membrane (IM), and outer membrane (OM) enriched fractions were separated based on Sarkosyl solubility and subjected to immunoblot analysis with antibodies against HA. Each fraction was concentrated to the same volume and equal volumes were loaded. (C) Immunoblot analysis of purified surface biotinylated proteins from lysates of SchuS4Δ*dipA* strains expressing HA-tagged DipA variants as described in (A). Input and biotinylated (surface) samples were processed for CFU enumeration and immunoblotting as described in Materials and Methods. Samples were loaded based on CFU equivalents as follows: 1x10^6^ (Input) or 1x10^8^ (surface). (D) Quantification of J774A.1 cells emitting blue fluorescence that were either uninfected or infected with SchuS4, or SchuS4 expressing C-terminal TEM1 fusions with IglI, IglA, DipA, DipAΔSel1ab, DipAΔSel1cd, DipAΔCC, DipACC(AIL ^3^D), or DipACC(LAL ^3^D). Infected cells were analyzed for CCF2/AM cleavage at 16 h pi. At least 100 cells were scored per experiment. Data are means ± SD from a representative experiment performed in triplicate out of three independent repeats. Asterisks indicate statistically significant differences compared to uninfected, SchuS4-infected, and SchuS4 expressing IglA-TEM1-infected controls (* *P < 0.001*, 1-way ANOVA, Tukey’s post-test). (E) Representative fluorescence micrographs of J774A.1 macrophages infected for 16 h with SchuS4 expressing either DipA-TEM1, DipAΔSel1ab-TEM1, DipAΔSel1cd-TEM1, DipAΔCC-TEM1, DipACC(AIL ^3^D)-TEM1, or DipACC(LAL ^3^D)-TEM1. Cells emitting blue fluorescence indicate delivery of TEM1 β-lactamase fusions to the cytosol to cleave the CCF2/AM substrate. Intact CCF2/AM, indicating the absence of TEM1 β-lactamase activity in the cytosol, results in green fluorescence emission. Scale bar, 50 µm.

Four regions of DipA were predicted to form putative SLR domains by SMART (residues 96-132, 133-169, 193-229, and 231-262) and Pfam (residues 100-132, 133-169, 196-229 and 235-262) (Figure 1A). SLRs are structural domains of paired anti-parallel α-helical repeats that provide a scaffold for protein–protein interactions [43]. SLR regions consist of variable sequence lengths spanning 36-44 amino acids with low sequence identity [43]. Because SLR sequences are highly divergent, we relied on homology model prediction by I-TASSER (C-Score -0.53, TM-Score 0.65 ± 0.13, RMSD 7.7 ± 4.3 Å) [44] to determine a mutagenesis strategy for the SLR regions of DipA (Figure 1C). The three dimensional structure prediction of DipA revealed a spatial proximity between the Sel1a and Sel1b helices, indicating potential interactions between these two SLR domains (Figure 1C). Similarly, the proximity of Sel1c and Sel1d domains suggests helical interactions between this pair of SLRs. Thus, we constructed two DipA SLR domain mutants according to this model: deletion of Sel1a and Sel1b between amino acid residues 95-170 (DipAΔSel1ab) and deletion of Sel1c and Sel1d between amino acid residues 192-263 (DipAΔSel1cd) (Figure 1A). HA-tagged versions of these DipA mutants were introduced into the SchuS4Δ*dipA* strain to evaluate the role of the SLR domains in DipA function. Expression of DipAΔSel1ab-HA and DipAΔSel1cd-HA mutants were similar to that of full-length DipA-HA (Figure S1A). In BMMs, SchuS4Δ*dipA* displayed limited intracellular growth whereas SchuS4 replicated 2.5 log over 16 h (Figure 4A). While the intracellular growth of SchuS4Δ*dipA* was restored to wild-type levels upon complementation with the full length DipA, neither DipAΔSel1ab nor DipAΔSel1cd significantly restored the ability of SchuS4Δ*dipA* to replicate in BMMs (Figure 4A). The DipAΔSel1ab and DipAΔSel1cd truncations partitioned like the full-length protein in the IM and OM fractions (Figure 4B), remained surface-exposed on SchuS4Δ*dipA* (Figure 4C), and were exposed to the macrophage cytosol during infection (Figure 4D and 4E). Thus, despite targeting to the correct location, the inability of either the DipAΔSel1ab or DipAΔSel1cd truncations to complement the SchuS4Δ*dipA* mutant growth defect indicates that the SLR domains are required for the biological function of DipA.

### The coiled-coil domain is required for DipA targeting to the bacterial surface

The putative CC domain of DipA was predicted by SMART (residues 311-343), COILS (residues 313-341) and Marcoil (residues 314-343) to form near the C-terminus (Figure 1A and 1C). CC domains are structural elements comprised of multiple amphipathic α-helices that wind around one another to generate distinct protein binding sites [45]. CC domain sequences characteristically contain a repetitive seven-residue pattern, represented as *a-b-c-d-e-f-g*, in which the residues at positions *a* and *d* form a hydrophobic core to drive inter-helical interactions [45]. The core residues of the DipA CC domain predicted by COILS to form the hydrophobic seam are L414, A317, L321, I324, and L331 (Figure 1B). Based on these predictions, we altered the putative CC domain by either in-frame deletion of the entire CC region between amino acid residues 311-343 (DipAΔCC, Figure 1A) or substitution of three key hydrophobic residues at position *d* of the α-helix interface to aspartate residues A317D, I324D, L331D [DipACC(AIL ^3^D)] or at positions *a* and *d* to aspartate residues L414D, A317D, L321D [DipACC(LAL ^3^D)] (Figure 1B). These three DipA CC mutants were compared to wild-type DipA in a SchuS4Δ*dipA* background to assess the role of the CC domain in DipA function. None of the DipA CC HA-tagged mutants were able to functionally complement the intracellular growth defect of SchuS4Δ*dipA* (Figure 4A), even though all three constructs were expressed at levels similar to full-length DipA-HA (Figure S1A). Although DipAΔCC-HA, DipACC(AIL ^3^D)-HA and DipACC(LAL ^3^D)-HA were detected in the IM and OM fractions of SchuS4Δ*dipA*, none were detected on the bacterial surface by biotinylation (Figure 4C). Moreover, the DipA CC mutants were not accessible to the macrophage cytosol as indicated by the low percentages of CCF2/AM conversion in macrophages infected with SchuS4 expressing DipAΔCC-TEM1 (5.1 ± 3.2%), DipACC(AIL ^3^D)-TEM1 (6.6 ± 1.4%) and DipACC(LAL ^3^D)-TEM1 (6.8 ± 1.1%), which were not significantly different from the negative controls (Figure 4D and 4E). Expression of these TEM1-fusions were also verified and showed little variation in expression levels compared with full length DipA-TEM1 (Figure S1B). Hence, the C-terminal coiled-coil domain of DipA is required for surface exposure of DipA. Moreover, these data indicate that proper surface localization of DipA is required to fulfill its function, although one cannot exclude that mutations of the CC domain functionally disable DipA independently of its localization.

### DipA interacts with FopA

**Figure 5:**
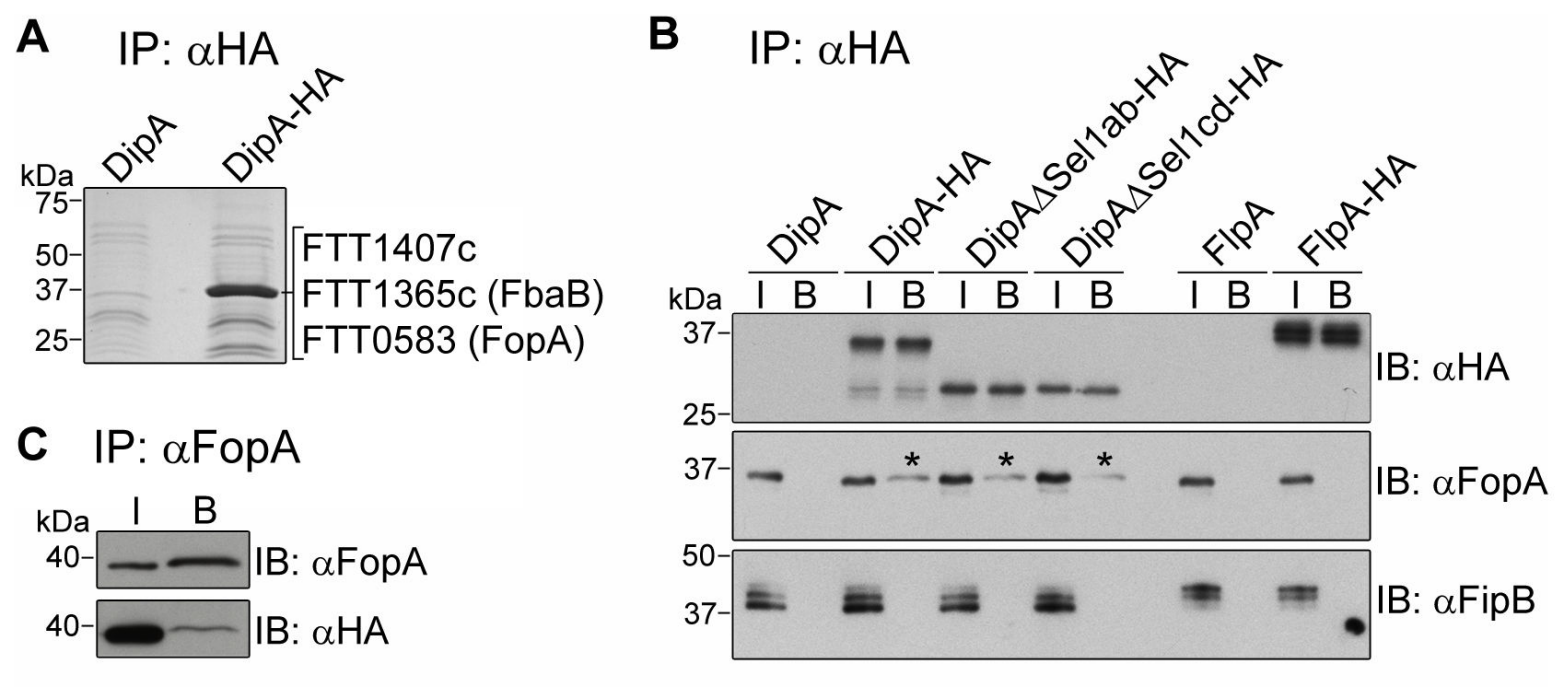
Identification of DipA interacting partners. (A) Code blue stained gel of proteins immunoprecipitated with anti-HA conjugated beads from lysates of SchuS4Δ*dipA* expressing DipA [SchuS4Δ*dipA*(p*dipA*)] or DipA-HA [SchuS4Δ*dipA*(p*dipA-HA*)]. Samples were resolved by SDS-PAGE and stained with GelCode® Blue. Protein bands migrating at ~40 kDa were subjected to mass spectrometry-based identification. FTT1407c, FTT1365c and FTT0583 were specifically co-immunoprecipitated with DipA-HA. (B and C) Verification of FopA interaction with DipA by immunoblot analysis. (B) Lysates from SchuS4Δ*dipA* expressing DipA derivatives (DipA, Dip-HA, DipAΔSel1ab-HA, DipAΔSel1cd-HA), or SchuS4Δ*flpA* expressing FlpA derivatives (FlpA, FlpA-HA) were subjected to immunoprecipitation (IP) using anti-HA conjugated beads followed by immunoblot (IB) analysis with anti-HA, anti-FopA and anti-FipB antibodies. I denotes sample input; B denotes bound fraction. (C) Lysate from SchuS4Δ*dipA* expressing DipA-HA was subjected to immunoprecipitation (IP) using anti-FopA antibodies followed by immunoblot (IB) analysis with anti-FopA antibodies to detect FopA and anti-HA antibodies to detect Dip-HA. I denotes sample input; B denotes bound fraction.

Having determined that DipA is exposed to the macrophage cytosol and contains a combination of domains known to mediate protein–protein interactions, we first hypothesized that DipA interacts with host factors to promote *Francisella* intracellular replication. To test this hypothesis, co-immunoprecipitation attempts using either ectopically expressed DipA-GFP in HeLa cells, pulldowns from HeLa and BMM cell lysates using purified recombinant DipA-GST, or co-immunoprecipitations from DipA-HA-expressing Schu S4-infected BMMs were performed, yet with no success (data not shown). We then hypothesized that DipA is part of a membrane-associated bacterial macromolecular complex and sought to identify potential bacterial interacting proteins by performing co-immunoprecipitation coupled to mass spectrometry. DipA-HA was immunoprecipitated from SchuS4Δ*dipA* expressing DipA-HA and additional proteins resolved by SDS-PAGE were identified by mass spectrometry. Peptides identified from an evident 40 kDa band co-immunoprecipitated with DipA-HA were compared to those identified from a faint corresponding band co-immunoprecipitated from SchuS4Δ*dipA* expressing non-tagged DipA to rule out non-specific candidates (Figure 5A). As expected, DipA was detected in the immunoprecipitated material from the lysate containing DipA-HA since it migrates at ~ 40 kDa. By this approach, we identified three *F. tularensis* proteins that were immunoprecipitated specifically along with DipA-HA: FTT1407c (16 matching peptides, 46% sequence coverage), a *Francisella* -specific putative membrane protein of unknown function; FTT1365c/FbaB (1 matching peptide, 3% sequence coverage), a fructose-1,6-bisphosphate aldolase (FBA) homolog; and FTT0583/FopA (1 matching peptide, 5% sequence coverage), an immunogenic outer membrane protein of unknown function (Figure 5A) [32,46]. Several glycolytic enzymes, including FBA, have been reported to localize to the surface of bacterial pathogens where they play a role in virulence [47–51]. Thus, the interacting protein candidates identified corroborate the localization profile of DipA as a surface-exposed membrane-associated protein.

Binding of DipA to FopA was confirmed by co-immunoprecipitation followed by immunoblot analysis with an anti-FopA antibody (Figure 5B). Due to a lack of reagents for FTT1407c and FbaB, we were unable to verify their interaction with DipA. FopA was specifically co-immunoprecipitated with DipA-HA, but not from control lysates of SchuS4Δ*dipA* expressing DipA lacking the HA-tag (Figure 5B). DipA interaction with FopA was confirmed by reciprocal co-immunoprecipitation (Figure 5C). To further examine the specificity of the DipA-FopA pull down, we tested whether FopA co-immunoprecipitated with FTT1676, a putative membrane lipoprotein [10]. Indeed, FTT1676 partitioned to the inner and outer membrane enriched fractions of GFP-expressing SchuS4 (Figure S2A), and was acylated in^3^ [H] palmitate incorporation experiments (Figure S2B), confirming the predicted membrane localization and lipoprotein nature of FTT1676. Hence, we renamed this protein FlpA for *Francisella* lipoprotein A. Unlike for DipA, FopA was not co-immunoprecipitated from lysates of SchuS4Δ*flpA* expressing either FlpA or HA-tagged FlpA, (Figure 5B). FipB, another *Francisella* lipoprotein, did not co-immunoprecipitate with DipA-HA, lending additional evidence to the specificity of FopA binding to DipA (Figure 5B) [30,31]. SLR domain mutations did not affect DipA interactions with FopA (Figure 5B), implying that other regions of DipA are responsible for this interaction. We were unable to assess the role of the CC domain in FopA binding because neither the HA-tagged nor FLAG-tagged versions of the DipA CC domain mutants could be efficiently immunoprecipitated with the corresponding antibodies (data not shown). In summary, these results demonstrate that DipA binds either directly or indirectly to FopA, an interaction that points toward a potential function of DipA as part of an outer membrane complex.

### Deletion of *fopA*, but not of FTT1407c, reproduces the intracellular defects of the ∆dipA mutant

**Figure 6:**
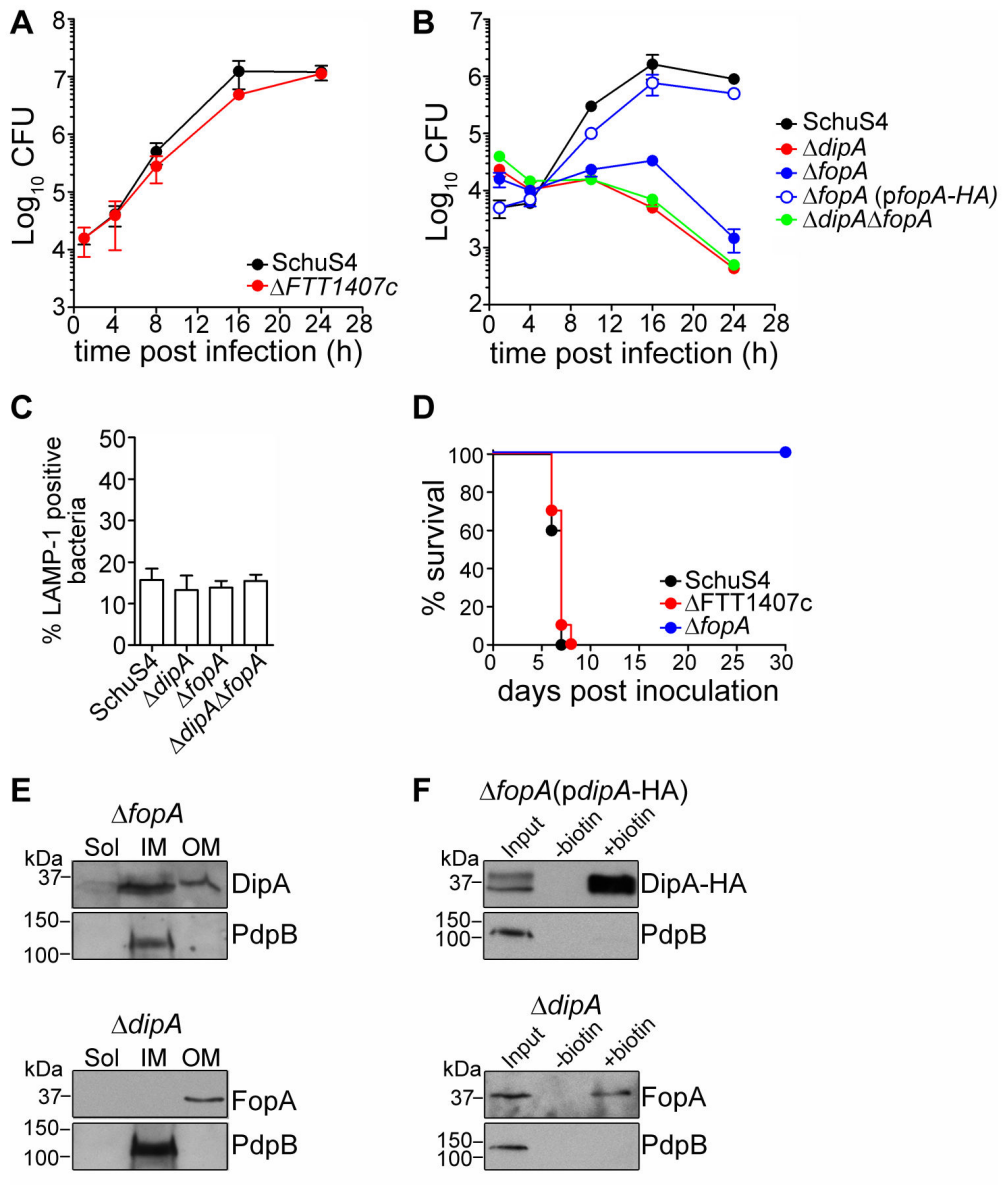
FopA is required for SchuS4 intracellular growth in BMMs and virulence in mice, but not for DipA outer membrane and surface localization. (A) Intracellular growth of SchuS4 and its isogenic ΔFTT1407c mutant in BMMs. BMMs were infected with either strain and CFUs were enumerated at various times p.i. Data are means ± SD from a representative experiment performed in triplicate out of two independent repeats. (B) Intracellular growth of SchuS4, its isogenic Δ*fopA* and Δ*dipAΔfopA* mutants and the complemented Δ*fopA*(p*fopA-HA*) strains in BMMs. BMMs were infected with either strain and CFUs were enumerated at various times p.i. Data are means ± SD from a representative experiment performed in triplicate out of two independent repeats. (C) Quantification of bacteria enclosed within LAMP-1-positive phagosomal membranes. BMMs were infected for 1 h with either SchuS4 or its isogenic Δ*dipA*, Δ*fopA* and Δ*dipAΔfopA* mutants. Samples were processed for immunofluorescence labelling of bacteria and LAMP-1-positive membranes. Infected BMMs were scored for number of infected cells with bacteria enclosed within LAMP-1-positive compartments. At least 100 bacteria per experiment were scored for each condition. Data are means ± SD from three independent experiments. (D) Survival curves of BALB/cJ mice infected with SchuS4, SchuS4ΔFTT1407c or SchuS4Δ*fopA* by intranasal inoculation. Intranasal inocula were 27 (SchuS4), 16 (ΔFTT1407c), and 15 (Δ*fopA*) CFUs. (E) Subcellular localization of DipA, FopA, and PdpB from SchuS4Δ*fopA* (top panels) or SchuS4Δ*dipA* (bottom panels). Soluble (Sol), inner membrane (IM), and outer membrane (OM) enriched fractions were separated based on Sarkosyl solubility and subjected to immunoblot analysis with antibodies against DipA, FopA, and PdpB. Each fraction was concentrated to the same volume and equal volumes were loaded. (F) Immunoblot analysis of purified surface biotinylated proteins from SchuS4Δ*fopA*(p*dipA-HA*) (top panels) or SchuS4Δ*dipA* (bottom panels) lysates. DipA-HA was detected using anti-HA antibodies; FopA was detected using anti-FopA antibodies. PdpB and was used as a negative control. Input, untreated (-biotin) and biotinylated (+biotin) samples were processed for CFU enumeration and immunoblotting as described in Materials and Methods. Samples were loaded based on CFU equivalents as follows: 2x10^6^ (Input) or 1x10^8^ (-/+ biotin) for anti-DipA-HA analysis, 5x10^6^ (Input) or 1x10^8^ (-/+ biotin) for anti-FopA analysis, 1x10^7^ (Input) or 5x10^8^ (-/+ biotin) for anti-PdpB analysis.

To extend our findings and explore the roles of the DipA interacting candidates in pathogenesis, we generated in-frame deletions of *fopA* and FTT1407c loci in SchuS4 and assessed the intracellular growth and *in vivo* virulence of the resulting mutants. Not surprisingly, since other enzymes in the glycolytic pathway are predicted to be essential [52], attempts to delete *fbaB* were unsuccessful, suggesting that this locus is indispensable for growth under *in vitro* conditions. Deletion of FTT1407c did not affect bacterial replication in BMMs over a period of 24 h (Figure 6A), whereas deletion of *fopA* resulted in impaired intracellular proliferation that mirrored the phenotype of SchuS4Δ*dipA* (Figure 6B) [10,53]. We then examined the intracellular trafficking of SchuS4Δ*fopA*, because phagosomal escape is a pre-requisite for cytosolic replication [9,10,26,54–56]. Using co-localization of bacteria with LAMP-1-positive membranes as an indicator of vacuolar or cytosolic location in BMMs, SchuS4Δ*fopA* showed phagosome escape kinetics similar to wild-type and SchuS4Δ*dipA* bacteria (Figure 6C). Thus, deletion of *fopA* specifically impairs cytosolic replication of SchuS4 in BMMs. Growth of the SchuS4Δ*fopA* mutant in modified Mueller-Hinton broth was not affected, excluding any physiological impairment (data not shown). *In trans* complementation with full-length *fopA* fully restored the ability of SchuS4Δ*fopA* to survive and grow in BMMs, confirming that the observed phenotypic defect was due to deletion of *fopA* (Figure 6B). Since SchuS4Δ*dipA* and SchuS4Δ*fopA* demonstrated similar intracellular growth defects, we examined the effect of a double deletion mutant of both loci on the ability of SchuS4 to replicate in BMMs, predicting that the combination of the two deletions should not have additive effects if both proteins participate in the same bacterial functions. SchuS4Δ*dipAΔfopA* bacteria showed the same intracellular defects as the single mutants (Figure 6B), further suggesting that DipA and FopA contribute to the same bacterial function that can be disabled by single deletion of either protein. We have shown previously that *dipA* is essential for SchuS4 virulence in BALB/cJ mice [10,57]. To test whether deletion of FTT1407c or *fopA* affects virulence of SchuS4, BALB/cJ mice were intranasally infected with 10 CFUs of each mutant strain. Similar to infection with wild-type SchuS4, all mice infected with the SchuS4ΔFTT1407c mutant had to be euthanized by 6 days p.i. (Figure 6D). In contrast, SchuS4Δ*fopA* displayed attenuated virulence, since 100% of intranasally infected animals survived up to 30 days p.i. (Figure 6D), consistent with the attenuation of the Δ*dipA* mutant [10].

Because both DipA and FopA are exposed on the surface of SchuS4 and interact with each other, we examined whether the absence of one affects the localization of the other. DipA remained enriched to the inner and outer membranes (Figure 6E) and its HA-tagged version was detected via biotinylation on the surface of a SchuS4Δ*fopA* strain (Figure 6F), thus, the localization of DipA does not depend on FopA. Reciprocally, localization of FopA to the bacterial outer membrane and exposure on the bacterial surface remained unaffected in the absence of DipA (Figure 6E and 6F). Hence, outer membrane localization and surface exposure of DipA and FopA are independent of one another.

In this study, we have examined how the various structural domains of DipA contribute to its functions, in order to gain insights into its role in *Francisella* pathogenesis. We demonstrate that DipA is targeted to the surface of SchuS4 to fulfill its role as a *Francisella* virulence factor. Our data highlights the importance of the SLR and CC domains for DipA function. Among other functions, proteins containing SLR domains have been found to serve as adaptor proteins for the assembly of membrane-bound macromolecular complexes. For example, the yeast Hrd3 SLR protein is anchored to the ER membrane in a complex that functions as part of the ER-associated protein degradation mechanism [58]. In prokaryotes, the SLR protein MotX from *Vibrio parahaemolyticus* is anchored to the cytoplasmic membrane via peptidoglycan and complexes with flagellar proteins, MotY and PomB, and contributes to motor function [59,60]. The effector proteins LpnE, EnhC, and LidL secreted by the *Legionella pneumophila* Type IV secretion system are SLR-containing proteins that have been shown to contribute to the trafficking of the *Legionella* containing vacuole and establishment of an intracellular replicative niche [61–63]. Deletion of even a pair of the eight SLR regions rendered LpnE non-functional, emphasizing the importance of these domains [63]. Similarly, deletion of either pair of the SLR domains impaired the ability of DipA to complement the growth defect of the SchuS4Δ*dipA* mutant. The CC domain is also important for DipA function. A key characteristic of CC domains is the proclivity to form multimeric complexes, a feature driven by structural stability [64,65]. For example, the majority of the 200 coiled-coil interactions from *Saccharomyces cerevisiae* involve multiple heterotypic partners [66]. In bacteria, CC domains are found commonly in proteins associated with secretion systems [67]. The CC domain of EspA, while dispensable for its secretion, is essential for the multimeric assembly of the type III secretion apparatus on the surface of Enteropathogenic *Escherichia coli* [68]. In *Salmonella enterica* serovar typhimurium, CC domains of T3SS effectors are responsible for their targeting to host membranes where they interact with host factors [69]. Given their structural nature and propensity for oligomerization, the presence of these domains suggests that DipA may function as a structural scaffold to link various components of a multiprotein complex that imparts a unique mechanism of virulence to *F. tularensis*.

Of the three putative DipA interacting partners identified, FopA may shed some light on the function of DipA even though the biological significance of the interaction is currently unclear. FopA contains a conserved OmpA domain at its C-terminus, while the rest of the protein sequence does not display homology to any known domains. The function of FopA has not been directly addressed; however, other OmpA-like proteins serve a myriad of functions including well-established roles in bacterial adhesion, invasion and immune evasion [70–74]. For pathogens such as uropathogenic *Escherichia coli* [75], *Neisseria gonorrhoeae* [76], and *Yersinia* spp. [77], their respective OmpA homologs have been shown to enhance intracellular survival. The OmpA-like protein of *Pseudomonas aeruginosa* OprF is thought to act as a sensor for virulence factor activation upon host cell contact [74]. We have also demonstrated a requirement for FopA in intramacrophage growth and *in vivo* virulence of SchuS4 (Figure 6). Additionally, OmpA homologs are also known to function structurally and in adaptation to environmental stresses [74,78–81]. Based on the demonstrated roles of various OmpA homologs, DipA may function as part of a structural membrane complex with FopA that contributes to the intracellular adaptation of *F. tularensis*, ultimately allowing it to survive and proliferate within the macrophage cytosol. Consistent with its contribution to the virulence of *F. tularensis*, DipA possesses domains and interactions with proteins that are implicated in bacterial pathogenesis. Further examination of these interactions will be important in discerning the role of these factors in the virulence of *F. tularensis*.

## Materials and Methods

### Ethics Statement

All animal rearing, handling and experimental methods were conducted in strict accordance with the recommendations in the Guide for the Care and Use of Laboratory Animals, 8th Edition, of the National Research Council under protocols approved by the Rocky Mountain Laboratories Institutional Animal Care and Use Committee (IACUC). The Rocky Mountain Laboratories is an AAALAC International-accredited institute.

### Bacterial strains and culture conditions

The prototypic Type A virulent strain, *F. tularensis* subsp. *tularensis* SchuS4 was obtained from Rick Lyons (University of New Mexico, Albuquerque, NM, USA). GFP-expressing SchuS4 and SchuS4∆*dipA* (∆FTT0369c) have been described previously [10]. SchuS4∆FTT1407c, SchuS4∆*fopA*, and SchuS4∆*dipA*∆*fopA* mutants were generated as described below. SchuS4 and its derivatives were grown either on modified Mueller-Hinton (MMH) agar plates [Mueller-Hinton medium supplemented with 0.1% glucose (Sigma), 0.025% ferric pyrophosphate (Sigma Aldrich) and 2% IsoVitaleX (Becton Dickinson) or MMH supplemented with 10 µg/ml kanamycin (Sigma) for 3 days at 37°C under 7% CO_2_. When indicated, colonies from freshly streaked MMH plates were re-suspended in MMH broth or Tryptic-Soy broth supplemented with 0.1% cysteine (TSB-C) grown at 37 °C under agitation. MMH medium was supplemented with either 10 µg/ml kanamycin (Sigma) or 8% sucrose (Sigma) for allelic replacement. All manipulations of *F. tularensis* strain SchuS4 and its derivatives were performed in a Biosafety Level 3 facility according to standard operating procedures approved by the Rocky Mountain Laboratories Institutional Biosafety Committee and the CDC Division of Select Agents and Toxins regulations.

### Sequence analysis and structural modelling

The translated amino acid sequence of DipA was analyzed using SMART (http://smart.embl-heidelberg.de/) and Pfam (http://pfam.sanger.ac.uk/) databases to identify conserved protein domains. SignalP (http://www.cbs.dtu.dk/services/SignalP/) and LipoP (http://www.cbs.dtu.dk/services/LipoP/) prediction programs were used to determine the signal peptidase cleavage site. COILS (http://embnet.vital-it.ch/software/COILS_form.html) [82] with windows of 28, 21 and 14 residues and Marcoil [83] were used in conjunction with SMART to predict the heptad repeat of the coiled-coil domain. A three-dimensional structure of DipA was built by the I-TASSER server (http://zhanglab.ccmb.med.umich.edu/I-TASSER/) [44,84] and visualized using the UCSF Chimera package (http://www.cgl.ucsf.edu/chimera) [85]. Chimera is developed by the Resource for Biocomputing, Visualization, and Informatics at the University of California, San Francisco (supported by NIGMS P41-GM103311). The model includes amino acid residues 21-353 of DipA.

### Plasmids and molecular techniques

Plasmids and primers used in this study are described in Tables S1 and S2, respectively. Plasmids used in this study were derived from pFNLTP6 [86]. pFNLTP6*omp26* (pJC900) was generated by amplification of the *omp26* promoter region from SchuS4 genomic DNA with primers JC801 and JC823; the amplified fragment was subcloned into the TA cloning vector pCR2.1 (Life Technologies) its sequence confirmed, and cloned into the *Eco*RI-digested vector pFNLTP6.

HA fusions were generated using primers described in Table S2. To tag DipA, FlpA, IglI, IglA, and FopA with double-HA, their open reading frames were PCR amplified using SchuS4 genomic DNA as template. The DNA fragment encoding the two tandem repeats of the HA tag was synthesized and used as a template, along with the PCR amplified open reading frames, in overlap extension PCR to fuse the two fragments. The *dipA-*HA and *flpA*-HA amplified fusions were sub-cloned into the TA cloning vector pCR2.1 and subsequently sequenced. The fusions were excised from pCR2.1 and ligated into the *Nhe*I/*Xho*I digested pFNLTP6 and pFNLTP6*omp26*, respectively. The *iglA-*HA and *fopA-*HA amplified fusions were cloned into the *Nhe*I/*Xho*I sites and the *iglI-*HA fusion was cloned into the *Not*I/*Xho*I sites of pFNLTP6*omp26* using the In-Fusion® PCR Cloning System. The Sel1 domain deletion mutants DipAΔSel1ab-HA and DipAΔSel1cd-HA were generated using the pFNLTP6-*dipA-*HA construct as template. To delete the first pair of SLR regions (DipAΔSel1ab), 300 bp upstream of the start codon, the start codon, and the first 285 bp of the *dipA* open reading frame were amplified with oligonucleotides JC943 and JC956; 552 bp of the *dipA* open reading frame downstream of the Sel1ab domain and the tandem HA tag were amplified with oligonucleotides JC955 and JC957. The two fragments were fused by overlap extension PCR and cloned into *Eco*RI- and *Xho*I-digested pFNLTP6 using In-Fusion® PCR Cloning System. To delete the second pair of SLR regions (DipAΔSel1cd), 300 bp upstream of the start codon, start codon, and first 573 bp of the DipA open reading frame were amplified with oligonucleotides JC943 and JC958; 273 bp of the *dipA* open reading frame downstream of the Sel1cd domain and the tandem HA tag were amplified with oligonucleotides JC955 and JC959. The two fragments were fused by overlap extension PCR and cloned into *Eco*RI- and *Xho*I-digested pFNLTP6 using In-Fusion® PCR Cloning System. To generate a CC deletion mutant of DipA with a tandem HA-tag, 300 bp upstream of the start codon, start codon, and first 927 bp of the *dipA* open reading frame were amplified with oligonucleotides JC943 and JC960 using pFNLTP6-*dipA-HA* as template. The 30 bp downstream of the CC domain and HA tag were amplified with oligonucleotides JC955 and JC961. The two fragments were fused by overlap extension PCR and cloned into *Eco*RI- and *Xho*I-digested pFNLTP6 using In-Fusion® PCR Cloning System. The CC domain substitution mutants, DipACC(AIL ^3^D)-HA and DipACC(LAL ^3^D)-HA, were generated by introducing the mutations within the primer sets used for overlap extension PCR and the pFNLTP6-*dipA-HA* construct as template. The resultant fragments were cloned into *Eco*RI- and *Xho*I-digested pFNLTP6 using In-Fusion® PCR Cloning System. The identity and orientation of all constructs generated by PCR were confirmed by restriction digest analysis, sequencing and protein expression (Figure S1A).

To tag DipA and IglI with TEM1, the open reading frames of these genes were PCR amplified using SchuS4 genomic DNA as template. The TEM1 tag was PCR amplified from template pCX340 [87]. The fragments were fused by overlap extension PCR and sub-cloned into pCR2.1. Following sequencing, *dipA*-TEM1 was excised and cloned into pFNLTP6*omp26* at *Nhe*I and *Xho*I sites. The *iglI*-TEM1 insert was excised and cloned into pFNLTP6*omp26* at *Not*I and *Xho*I sites. The *iglA*-TEM1 insert was cloned directly into pFNLTP6*omp26* at *Nhe*I and *Xho*I sites by In-Fusion® PCR Cloning System. The DipA domain deletions were TEM1 tagged by PCR-amplifying the domain deletions using previously constructed pFNLTP6 HA-tagged constructs, and fused with TEM1 by overlap extension using primers described in Table S2. The identity and orientation of all constructs generated by PCR were confirmed by restriction digest analysis, sequencing and protein expression (Figure S1B).

### Construction of SchuS4 deletion mutants

SchuS4 in-frame deletion mutants were generated using the SacB-assisted allelic replacement suicide vector pJC84, as described previously [10]. Deletions of either the *fopA*, FTT1407c or *fbaB* loci were designed to preserve the integrity of downstream genes and avoid any polar effects. To engineer an in-frame deletion of *fopA*, a 5’-fragment containing 1253 bp upstream of the start codon of *fopA* (FTT0583), its start and the first codon was generated by PCR amplification using the primers TW202 and TW203, and a 3’ fragment containing 971 bp downstream of the stop codon and the last 7 codons of *fopA* using primers TW204 and TW205 (Table S2). The fragments were fused by overlap extension PCR using primers TW202 and TW205. The resulting fragment was cloned into the *Sal*I and BglII sites of pJC84 using the In-Fusion® PCR Cloning System (Clontech) and was fully sequenced. To engineer an in-frame deletion of FTT1407c, a 5’-fragment containing 969 bp upstream of the start codon of FTT1407c, its start and the first codon was generated by PCR amplification using the primers TW151 and TW152, and a 3’ fragment containing 1116 bp downstream of the stop codon and the last 6 codons of FTT1407c using primers TW153 and TW154 (Table S2). The fragments were fused by overlap extension PCR using primers TW151 and TW154. The resulting fragment was cloned into the BamHI and *Sal*I sites of pJC84 using the In-Fusion® PCR Cloning System (Clontech) and was fully sequenced. To engineer an in-frame deletion of *fbaB*, a 5’-fragment containing 1476 bp upstream of the start codon of *fbaB* (FTT1365c), its start and the first 3 codons was generated by PCR amplification using the primers RC407 and RC408, and a 3’ fragment containing 1385 bp downstream of the stop codon and the last 8 codons of *fopA* using primers RC409 and RC410 (Table S2). The fragments were fused by overlap extension PCR using primers RC407 and RC410. The resulting fragment was cloned into the BamHI and *Sal*I sites of pJC84 using the In-Fusion® PCR Cloning System (Clontech) and was fully sequenced.

To perform allelic replacement in the chromosome of Schu S4, electrocompetent bacteria were prepared and electroporated with recombinant pJC84 plasmid DNA as previously described [10]. Kanamycin-resistant merodiploid colonies were tested for integration of the allelic replacement plasmid, using colony PCR with primers JC420 and JC427 or JC589 and JC428 (Table S2). Independent clones were then subjected to sucrose counter selection to isolate clones that have undergone allelic replacement as previously described [10]. Absence of the targeted allele and allelic replacement within the correct chromosomal locus were verified by PCR using primers listed in Table S2. Independent clones with the correct in-frame deletion were isolated and used for further studies. Allelic replacement was performed on the single deletion mutant Δd*ipA* to generate the double deletion mutant Δ*dipAΔfopA*.

### Antibody generation

The rat polyclonal anti-DipA and anti-FlpA antisera used in this study were generated by Aldevron (Freiburg, Germany) via genetic immunization using codon-optimized FTT0369c and FTT1676 open reading frames. Rabbit polyclonal antisera against PdpB and IglA were generated by New England Peptide, Inc. by immunizing New-Zealand rabbits with PdpB (KTDDRWESKDFSKPEC) and IglA (CSVDAKKEFADREVRR) peptides.

### Immunoblot analysis

Samples were resolved on 12% SDS-polyacrylamide gels, and transferred to Hybond-ECL nitrocellulose membranes (Amersham Biosciences) as previously described [9]. Primary antibodies used for immunoblot analysis were: mouse monoclonal anti-HA (clone 16B12, Covance Research Products), rat anti-DipA (this study), rat anti-FopA (gift from Jason Huntley, University of Toledo, Toledo, Ohio), rabbit anti-PdpB (this study), rabbit anti-IglA (this study), rabbit anti-GFP (Life Technologies), rabbit anti-FipB [31], rat anti-FlpA (this study). Secondary antibodies used were: HRP-conjugated anti-rabbit IgG, anti-rat IgG or anti-mouse IgG (Cell Signaling Technology), and HRP-conjugated light chain specific anti-rat IgG or anti-mouse IgG (Jackson ImmunoResearch). Immunodetection was performed using SNAP i.d. (Millipore). Signals were visualized with ECL Advance™ immunoblotting detection kit (Amersham Biosciences) followed by autoradiography and scanning using a Kodak Image Station 4000MM Pro and assembled using Adobe Photoshop CS3.

### Fractionation

For fractionation of SchuS4 strains, ~2x10^11^ CFU were harvested from freshly streaked MMH plates, washed once with PBS, and resuspended in ice cold lysis buffer [PBS supplemented with EDTA-free cOmplete protease inhibitor tablet (Roche), 0.6 µg/mL DNase I (Sigma) and 0.6 µg/mL RNase (Roche)]. Aliquots of these suspensions were taken for plating to determine the actual CFU. Bacteria were resuspended into Lysing Matrix B tubes containing 0.1 mm silica spheres (MP Biomedicals) and disrupted for 9-cycles of 45 s/cycle at maximum speed in a FastPrep FP120 Cell Disrupter (ThermoSavant). Tubes were incubated on ice for 1.5 min between cycles. Samples were taken for plating to determine lysis. Lysates were clarified by two rounds of centrifugation at 8000 x *g* at 4 °C for 10 min followed by ultracentrifugation for 1.5 h at 100 000 x *g* at 4 °C. Supernatants were saved as soluble protein fractions. Membrane pellets were resuspended in 1% Sarkosyl (Sigma) and subjected to ultracentrifugation for 1.5 h at 100 000 x *g* at 4 °C to separate the Sarkosyl-soluble (inner membrane) and Sarkosyl-insoluble (outer membrane) fractions. Each fraction was ultracentrifuged twice to ensure efficient separation. The pelleted outer membrane fractions were resuspended in PBS. Soluble and inner membrane fractions were concentrated using 10 kDa cut-off Amicon ultrafilters (Millipore) to the same volume as the outer membrane fractions. Equal volumes from each fraction were loaded and separated by SDS-PAGE followed by immunoblotting analysis.

### Biotinylation of SchuS4 surface proteins

SchuS4 strains were harvested from freshly streak MMH plates, washed three times by centrifugation at 10,000 x *g* at 4 °C for 5 min, and resuspended in cold PBS at ~1x10^9^ CFU/mL. Aliquots (0.5 mL) were incubated with 0.25 mL of EZ-Link sulfo NHS-LC-LC-biotin (15 mg/mL, Pierce) for 0.5 h at room temperature to label surface proteins. Biotinylated suspensions of intact bacteria were centrifuged at 8000 x *g* at 4 °C for 4 min, washed with 1 mL of a salt solution (50 mM Tris, 300 mM NaCl, pH 7.5) and two 1 mL washes with cold PBS. Aliquots were taken for plating to determine the actual CFU. After the final wash, bacteria were resuspended in 50 µL of PBS, lysed with 500 µL of B-PERII (Pierce), aliquots plated to ensure complete lysis, and centrifuged at 15,200 x *g* at 4 °C for 1 min to clarify the lysate. Supernatants were transferred to new tubes to which 0.2 mL of Ultralink immobilized NeutrAvidin beads (Pierce) were added. Samples were incubated for 30 min at room temperature with gentle rocking. The bead complexes were washed thrice in 1 mL of high salt buffer [500 mM NaCl, 25 mM Tris, pH 7.5, 0.2% Tween 20 (v/v)] followed by two more washes in 1 mL of low salt buffer [50 mM NaCl, 25 mM Tris, pH 7.5, 0.2% Tween 20 (v/v)] using centrifugation conditions of 1000 x *g* for 1 min at room temperature. To recover biotinylated proteins, pelleted NeutrAvidin beads were resuspended in 40 µL of standard SDS-PAGE sample buffer, boiled at 95 °C for 15 min, centrifuged at 500 x *g* for 1 min to separate supernatants containing biotinylated proteins from the beads. The supernatants were normalized to CFU equivalents resolved by SDS-PAGE and subjected to immunoblot analysis.

### Macrophage culture, infection and bacterial CFU determination

Murine BMMs harvested from C57BL/6J (Jackson Laboratories) were differentiated as described previously [9]. J774A.1 macrophage-like cells (ATCC, TIB-67) were cultured and maintained in 4.5 g/L glucose Dulbecco’s Modified Eagle Medium (DMEM, Life Technologies) supplemented with 10% fetal bovine serum (FBS, Life Technologies) and 4 mM L-glutamine (Life Technologies). Two days prior to infection, J774A.1 cells were plated onto 96-well flat cell culture-treated plates at a density of 1x10^4^ macrophages/well. Immediately prior to infection of macrophages, a few colonies from a freshly streaked plate were resuspended in MMH broth and the OD_600nm_ was measured to estimate bacterial numbers. Bacteria were diluted to the appropriate multiplicity of infection (MOI) in BMM media and 0.5 ml of bacterial suspension was added to chilled cells. Macrophage were infected at an applied MOI of 50 (BMM) or 100 (J774A.1) as previously described [9]. Intracellular growth was monitored by determining the number of colony-forming units (CFU) recovered from lysed BMMs as previously described [9]. At 1 h and 16 h p.i., the numbers of viable intracellular bacteria from lysed BMMs were determined from triplicate wells.

### Immunofluorescence microscopy

BMMs on 12 mm glass coverslips were infected as described previously [9]. To ensure only BMM plasma membranes were permeabilized, infected BMMs were washed three times with PBS, fixed for 10 min at 37 °C in 1% paraformaldehyde, washed three times with PBS, and incubated for 10 min in 50 mM NH_4_Cl in PBS to quench free aldehyde groups. Samples were blocked and permeabilized in blocking buffer (10% horse serum, 0.05% saponin in PBS) for 30 min at room temperature. Samples were labelled by incubating inverted coverslips on drops of primary antibodies diluted in blocking buffer for 1 h at room temperature. Primary antibodies used were mouse monoclonal anti-*F. tularensis* LPS (US Biologicals), rat monoclonal anti-HA (clone 3F10, Life Technologies) and rabbit anti-GFP (Life Technologies). Bound antibodies were detected by incubation of coverslips on drops of secondary antibodies diluted in blocking buffer for 1 h at room temperature. Secondary antibodies used were Alexa Fluor™ 568-conjugated goat anti-mouse (Life Technologies), Alexa Fluor™ 568-conjugated goat anti-rabbit (Life Technologies) and Alexa Fluor™ 488-conjugated goat anti-rabbit (Life Technologies). Macrophage nuclei were counterstained with DAPI (Life Technologies) diluted in ddH _2_0 for 10 min at room temperature after incubation with secondary antibodies. Samples were washed twice with 0.05% saponin in PBS, once in PBS and once in ddH _2_0 and then mounted in Mowiol 4-88 mounting medium (Calbiochem). When permeabilized and stained under these conditions, antibody labelling of GFP was detected in fewer than 5% of BMMs infected with GFP-expressing SchuS4 (data not shown). Labelling of GFP by antibodies was readily detected in BMMs infected with GFP-expressing SchuS4 when permeabilized with 0.5% Triton X-100 (Sigma). *In vitro* grown bacteria were immunostained in suspension as described above and mounted on slides in Mowiol 4-88. Confocal images of 1024x1024 pixels were acquired using Carl Zeiss LSM 710 confocal laser scanning microscope for image acquisition and assembled using Adobe Photoshop CS3.

### TEM1 β-lactamase assay

TEM1 fusions described in Table S1 were electroporated into SchuS4 and expression verified by immunoblot analysis with an anti-β-lactamase antibody (QED Bioscience Inc). J774.A1 cells were then infected with SchuS4 strains harboring the TEM1 fusions at an MOI of 100 as previously described [9]. At 16 h pi, cells were washed twice with PBS and loaded with the fluorescent substrate CCF2/AM (LiveBLAzer-FRET B/G loading kit, Life Technologies) in the β-lactamase loading solution supplemented with 15 mM Probenecid (Life Technologies). Cells were incubated in the dark for 90 min at room temperature and then observed under epifluorescence using a Carl Zeiss Axiovert 200M fitted with a β-lactamase Blue/Aqua 41031 filterset (Chroma Technology Corp.). At least 300 cells were counted in triplicate wells to determine the percentage of cells emitting blue fluorescence (TEM1-positive). Data are means + SD from three independent experiments performed in triplicate. Following quantification, macrophages were then fixed and immunostained for *Francisella* with a mouse monoclonal anti-*F. tularensis* LPS (US Biologicals) antibody to verify that > 95% cells were infected, ensuring that the CCF2/AM cleavage occurred in infected cells.

### Co-immunoprecipitations

Co-immunoprecipitations to identify potential DipA interacting protein partners were performed with agarose beads conjugated to monoclonal anti-HA antibody (Clone HA-7, Sigma). Approximately 10 OD_600_ of SchuS4Δ*dipA* expressing either DipA [SchuS4Δ*dipA*(pFNLTP6*dipA*)] or DipA-HA [SchuS4Δ*dipA*(pFNLTP6*dipA*-HA)] were harvested from overnight MMH broth cultures. The cultures were washed twice with cold PBS and lysed with 1 mL/5 OD_600_ of B-PERII lysis reagent (Pierce). The lysates were then clarified by three centrifugations at 16,100 x *g* at 18 °C for 10 min, then the supernatants were incubated with anti-HA conjugated beads for 3 h at room temperature with gentle rotation. The bead complexes were washed three times with B-PERII then twice with wash buffer (0.2% NP40, 150 mM NaCl, 1 mM MgCl_2_, 20 mM Tris, pH 7.5) in which the mixtures were centrifuged at 8,200 x *g* for 1 min. Immunoprecipitated proteins were recovered from the beads with three rounds of 50 µL elution buffer (0.1 M glycine, pH 2.5, 0.2% NP40), neutralized with 15 µL Tris pH 9.5, and concentrated with Amicon Ultrafilters (10 kDa cut-off, Millipore). Immunoprecipitated proteins were resolved by SDS-PAGE and visualized with GelCode® Blue Stain Reagent (Thermo Scientific). A prominent band of proteins with apparent molecular weight of 40 kDa co-immunoprecipitated from SchuS4Δ*dipA*(pFNLTP6*dipA*-HA) lysate was excised and analyzed by mass spectrometry by the Taplin Biological Mass Spectrometry Facility (Harvard Medical School, Boston, MA). A corresponding band of proteins co-immunoprecipitated from SchuS4Δ*dipA*(pFNLTP6*dipA*) lysate was similarly analyzed to serve as a background control. Only peptides identified uniquely by co-immunoprecipitation from SchuS4Δ*dipA*(pFNLTP6*dipA*-HA) lysate were considered potential DipA interacting candidates; common peptides identified from both co-immunoprecipitated samples were disregarded.

FopA interaction with DipA was verified by co-immunoprecipitation using the bead conjugated anti-HA antibody as described above using 1 OD_600_ of SchuS4Δ*dipA*(pFNLTP6*dipA*), SchuS4Δ*dipA*(pFNLTP6*dipA*-HA), SchuS4Δ*dipA*(pFNLTP6*dipAΔSel1ab*-HA), SchuS4Δ*dipA*(pFNLTP6*dipAΔSel1cd*-HA), SchuS4Δ*flpA* (*p*FNLTP6*omp26-flpA*), or SchuS4Δ*flpA* (pFNLTP6*omp26-flpA*-HA) cultures. Immunoprecipitated material was resolved by SDS-PAGE and analyzed by immunoblotting for HA-tagged, FopA and FipB proteins.

FopA interaction with DipA was also confirmed by reverse co-immunoprecipitation using antibodies against FopA (gift from Jason Huntley) coupled to Dynabeads® Protein G (Life Technologies). To couple antibody to beads, 2% anti-FopA antibodies (v/v) in PBS were incubated with Dynabeads® Protein G for 1 h at room temperature with gentle agitation, washed three times with PBS, and resuspended in 1 mL of PBS. Approximately 1 OD_600_ of SchuS4Δ*dipA*(pFNLTP6*dipAHA*) lysate, prepared for co-immunoprecipitation as described above, was incubated with anti-FopA antibody-coupled protein G beads for 3 h at room temperature. The bead complexes were washed five times in PBS using Dynal® MPC^TM^ magnet (Life Technologies). Co-immunoprecipitated proteins were separated from the beads by boiling for 10 min in SDS-PAGE loading buffer and centrifugation at 16,000 x *g* for 5 min. The supernatants containing co-immunoprecipitated proteins were resolved by SDS-PAGE and analyzed by immunoblotting for FopA and HA-tagged DipA. Light chain specific HRP conjugated secondary antibodies (Jackson ImmunoResearch) were used for protein detection to minimize interference from the precipitated primary antibody.

To [^3^H] palmityolate and immunoprecipitate FlpA, a fresh overnight culture of SchuS4 was subcultured into TSB-C broth and incubated until 0.3 OD_600_ at which point either 200 µCi/mL of [^3^H] palmitic acid or an equivalent volume of ethanol was added and incubated overnight. Approximately 1 OD_600_ of radiolabelled or unlabeled SchuS4 culture was harvested, washed once with PBS, and lysed with 0.5 mL of RIPA buffer (Sigma). The clarified lysates were agitated overnight at 4 °C with rat anti-FlpA coupled Dynabeads® Protein G (Life Technologies). Antiserum coupled-beads were prepared as described above. The bead complexes were washed five times in PBS using Dynal® MPC^TM^ magnet (Life Technologies). Co-immunoprecipitated proteins were separated from the beads by boiling for 5 min in SDS-PAGE loading buffer and centrifugation at 16,1000 x *g* for 5 min. Supernatants containing co-immunoprecipitated proteins were resolved by SDS-PAGE, transferred onto Immobilon™-P PVDF membrane (Sigma-Aldrich), and analyzed either by autoradiography or by immunoblot analysis with anti-FlpA antibodies.

### Animal infections

Groups of ten 6-8 week old BALB/cJ mice were intranasally infected with the indicated wild-type or mutant SchuS4 strains for survival studies as previously described [10]. Briefly, mice were anesthetized intraperitoneally with 12.5 mg/mL ketamine and 3.8 mg/mL xylazine immediately prior to infection. Bacteria were diluted in PBS and inoculated into the nares of each mouse in 25 µL total volume. Actual doses were determined by plating onto MMH agar plates. All infections were performed in an Animal Biosafety Level 3 (ABSL3) facility according to protocols reviewed and approved by the Rocky Mountain Laboratories Institutional Biosafety Committee and the RML Institutional Animal Care and Use Committee (IACUC), in compliance with the CDC Division of Select Agents and Toxins regulations.

## Supporting Information

Figure S1 Expression of HA- and TEM1-fusion proteins by *F. tularensis*. (A) Immnoblot analysis of C-terminally tagged HA-fusion proteins. Sample loading was normalized to 1x10^6^ CFU/lane from lysates of SchuS4 expressing IglA-HA or IglI-HA, SchuS4Δ*dipA* expressing DipA-HA, DipAΔSel1ab-HA, DipAΔSel1cd-HA, DipAΔCC-HA, DipA-CC(AIL ^3^D)-HA, or DipA-CC(LAL ^3^D)-HA, SchuS4Δ*flpA* expressing FlpA-HA, and SchuS4Δ*fopA* expressing FopA-HA. (B) Immnoblot analysis of C-terminally tagged TEM1-fusion proteins. Sample loading was normalized to 5x10^6^ CFU/lane from lysates of SchuS4 and SchuS4 expressing DipA-TEM1, DipAΔSel1ab-TEM1, DipAΔSel1cd-TEM1, DipA-CC(AIL ^3^D)-TEM1, DipA-CC(LAL ^3^D)-TEM1, DipAΔCC-TEM1, IglA-TEM1 or IglI-TEM1. Samples were resolved by SDS-PAGE and analyzed by immunoblot analysis with anti-HA (A) or anti-TEM1 β-lactamase (B) antibodies.
(TIF)

Figure S2 FlpA is a membrane lipoprotein. (A) Subcellular localization of FlpA from GFP-expressing SchuS4. Soluble (Sol), inner membrane (IM), and outer membrane (OM) enriched fractions were separated based on Sarkosyl solubility and subjected to immunoblot analysis with antibodies against FlpA. Each fraction was concentrated to the same volume and equal volumes were loaded. (B) Autoradiograph of [^3^H] palmitate radiolabeled SchuS4 lysate (lane 1), and unlabeled (lane 2) or [^3^H] palmitate radiolabeled (lane 3) lysates that were subjected to immunoprecipitation with anti-FlpA antibodies. Samples were separated by SDS-PAGE, and analyzed by autoradiography. (C) Immunoblot analysis of the same samples as (B) probed with anti-FlpA antibodies. FlpA, IgG heavy (IgG Hc) and light (IgG Lc) chain bands are indicated by arrows.
(TIF)

Table S1 Plasmids used in this study. (DOCX)

Table S2 Primers used in this study. (DOCX)
