# Supplementary figures and images for: Structure-Function Analysis of DipA, a Francisella tularensis Virulence Factor Required for Intracellular Replication
Source: PLoS One. 2013 Jun 26;8(6):e67965. doi: 10.1371/journal.pone.0067965 (PMC3694160; doi:10.1371/journal.pone.0067965)

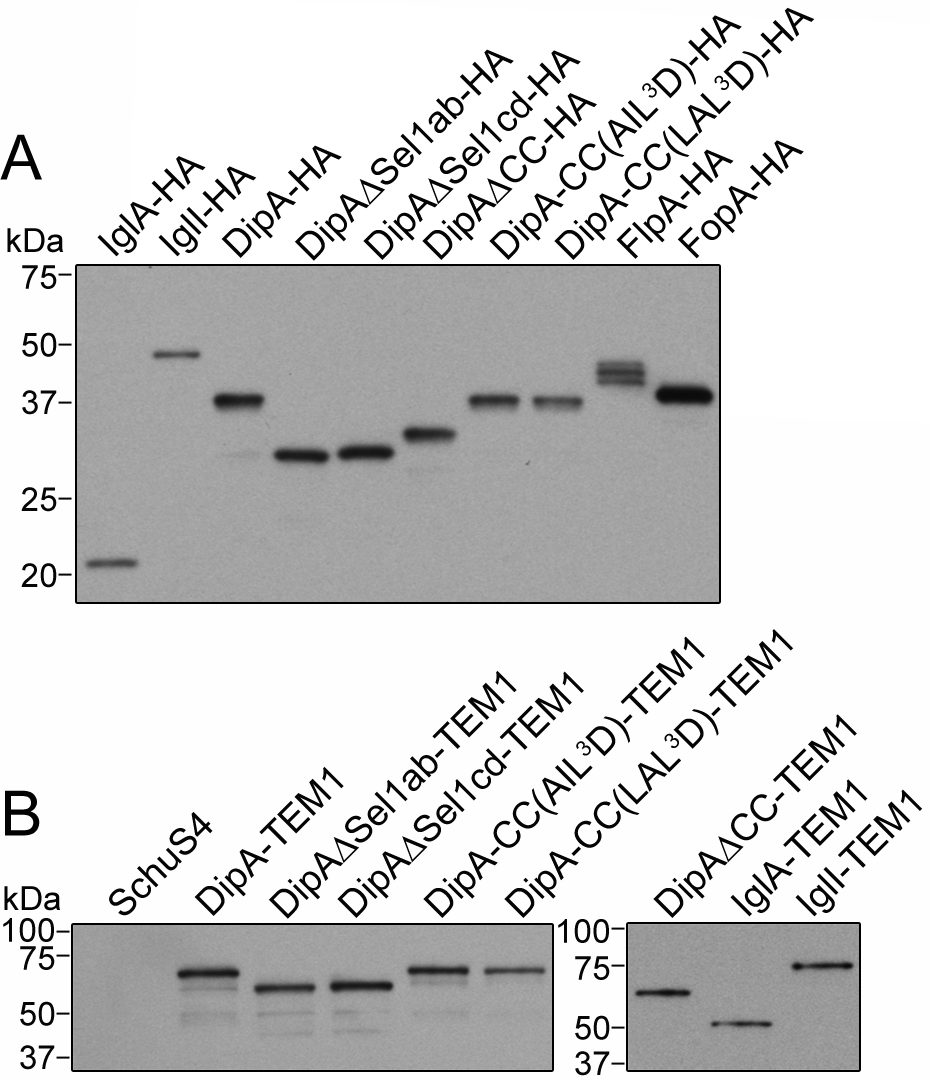

Supplement: Figure S1 — (A) Immnoblot analysis of C-terminally tagged HA-fusion proteins. Sample loading was normalized to 1x10^6^ CFU/lane from lysates of SchuS4 expressing IglA-HA or IglI-HA, SchuS4ΔdipA expressing DipA-HA, DipAΔSel1ab-HA, DipAΔSel1cd-HA, DipAΔCC-HA, DipA-CC(AIL 3D)-HA, or DipA-CC(LAL 3D)-HA, SchuS4ΔflpA expressing FlpA-HA, and SchuS4ΔfopA expressing FopA-HA. (B) Immnoblot analysis of C-terminally tagged TEM1-fusion proteins. Sample loading was normalized to 5x10^6^ CFU/lane from lysates of SchuS4 and SchuS4 expressing DipA-TEM1, DipAΔSel1ab-TEM1, DipAΔSel1cd-TEM1, DipA-CC(AIL 3D)-TEM1, DipA-CC(LAL 3D)-TEM1, DipAΔCC-TEM1, IglA-TEM1 or IglI-TEM1. Samples were resolved by SDS-PAGE and analyzed by immunoblot analysis with anti-HA (A) or anti-TEM1 β-lactamase (B) antibodies. (TIF) [file pone.0067965.s001.tif]

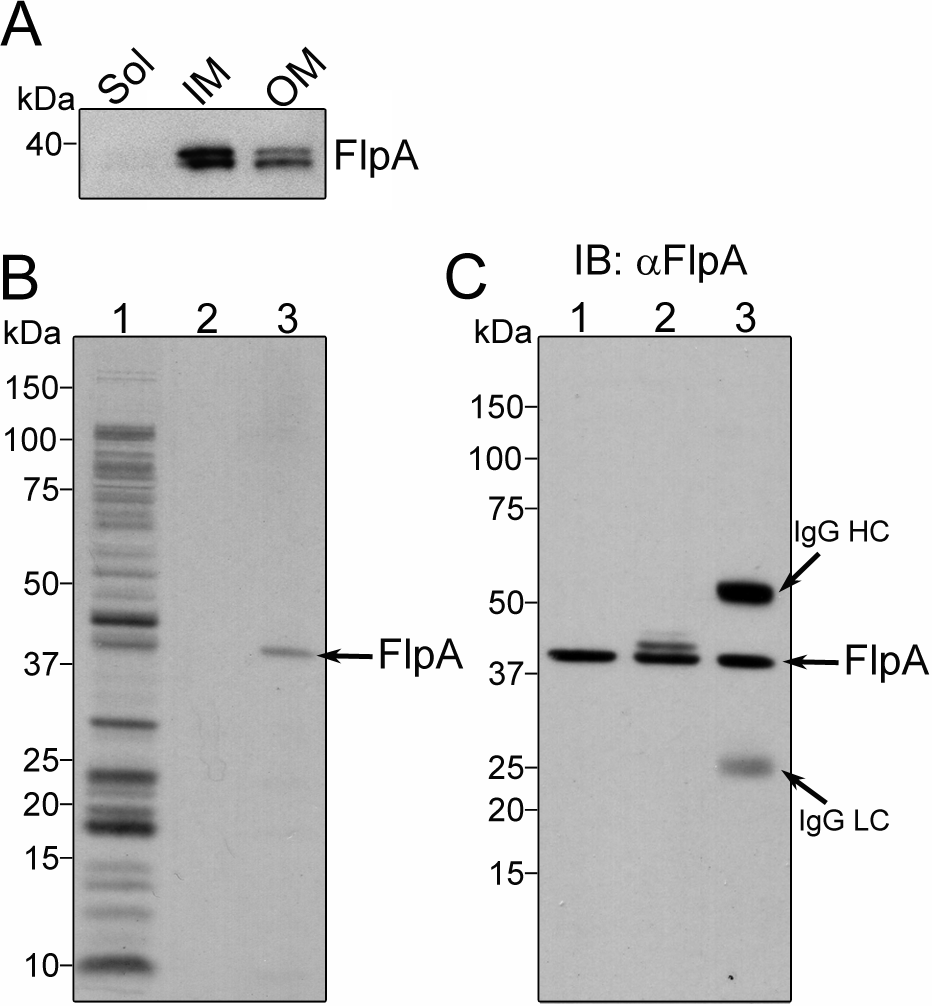

Supplement: Figure S2 — (A) Subcellular localization of FlpA from GFP-expressing SchuS4. Soluble (Sol), inner membrane (IM), and outer membrane (OM) enriched fractions were separated based on Sarkosyl solubility and subjected to immunoblot analysis with antibodies against FlpA. Each fraction was concentrated to the same volume and equal volumes were loaded. (B) Autoradiograph of [3H] palmitate radiolabeled SchuS4 lysate (lane 1), and unlabeled (lane 2) or [3H] palmitate radiolabeled (lane 3) lysates that were subjected to immunoprecipitation with anti-FlpA antibodies. Samples were separated by SDS-PAGE, and analyzed by autoradiography. (C) Immunoblot analysis of the same samples as (B) probed with anti-FlpA antibodies. FlpA, IgG heavy (IgG Hc) and light (IgG Lc) chain bands are indicated by arrows. (TIF) [file pone.0067965.s002.tif]
