# Supplementary material for: Structure-Function Analysis of DipA, a Francisella tularensis Virulence Factor Required for Intracellular Replication
Source: PLoS One. 2013 Jun 26;8(6):e67965. doi: 10.1371/journal.pone.0067965 (PMC3694160; doi:10.1371/journal.pone.0067965)
Supplement: Table S1 — (DOCX) [file pone.0067965.s003.docx]

Table S1. Plasmids used in this study.

| **Plasmid** | **Description/Genotype** | **Source** |
| --- | --- | --- |
| pFNLTP6 | *E. coli*-*F. tularensis* shuttle vector with NdeI, EcoRI, SmaI, NotI, NheI, and XhoI restriction enzyme sites (MCS2) cloned between the KpnI and BamHI sites; Km^r^ Ap^r^ | [[1](#_ENREF_1)] |
| pFNLTP6omp26 (pJC900) | derivative of pFNLTP6 with omp26 promoter region | This study |
| pFNLTP6*groE*-*gfp* | *gfp* under the control of the *groE* promoter region | [[1](#_ENREF_1)] |
| pCX340 | pBRR322 derivative containing TEM1 β-lactamase gene | [[2](#_ENREF_2)] |
| pFNLTP6*dipA* (pJC903) | *dipA* under the control of its own promoter region | [[3](#_ENREF_3)] |
| pFNLTP6*flpA* (pJC904) | *flpA* (*FTT1676)* under the control of the *omp26* promoter region | [[3](#_ENREF_3)] |
| pFNLTP6*dipA-HA* | fusion of *dipA* to 2X HA-tag under the control of the *dipA* promoter region | This study |
| pFNLTP6*omp26*-*iglA-HA* | fusion of *iglA* to 2X HA-tag under the control of the *omp26* promoter region | This study |
| pFNLTP6*omp26*-*iglI-HA* | fusion of *iglI* to 2X HA-tag under the control of the *omp26* promoter region | This study |
| pFNLTP6*dipAΔSel1ab-HA* | Residues 1-95, 170-353 of *dipA* fused to 2X HA-tag under the control of the *dipA* promoter region | This study |
| pFNLTP6*dipAΔSel1cd-HA* | Residues 1-192, 263-353 of *dipA* fused to 2X HA-tag under the control of the *dipA* promoter region | This study |
| pFNLTP6*dipAΔCC-HA* | Residues 1-311 of *dipA* fused to 2X HA-tag under the control of the *dipA* promoter region | This study |
| pFNLTP6*dipACC(AIL^3^D)-HA* | *dipA* coiled-coil domain mutant A317D, I324D, L331D fused to 2X HA-tag under the control of the *dipA* promoter region | This study |
| pFNLTP6*dipACC(LAL^3^D)-HA* | *dipA* coiled-coil domain mutant L314D, A317D, L321D fused to 2X HA-tag under the control of the *dipA* promoter region | This study |
| pFNLTP6*omp26*-*flpA*-HA | fusion of *flpA* to 2X HA-tag under the control of the *omp26* promoter region | This study |
| pFNLTP6*omp26*-*fopA*-HA | fusion of *fopA* to 2X HA-tag under the control of the *omp26* promoter region | This study |
| pFNLTP6*omp26*-*dipA-TEM1* | fusion of *dipA* fused to TEM1 under the control of the *omp26* promoter region | This study |
| pFNLTP6*omp26*-*iglA-TEM1* | fusion of *iglA* fused to TEM1 under the control of the omp26 promoter region | This study |
| pFNLTP6*omp26*-*iglI-TEM1* | fusion of *iglI* fused to TEM1 under the control of the *omp26* promoter region | This study |
| pFNLTP6*omp26-dipAΔSel1ab-TEM1* | Residues 1-95, 170-353 of *dipA* fused to TEM1 under the control of the *omp26* promoter region | This study |
| pFNLTP6*omp26-dipAΔSel1cd-TEM1* | Residues 1-192, 263-353 of *dipA* fused to TEM1 under the control of the *omp26* promoter region | This study |
| pFNLTP6*omp26*-*dipAΔCC-TEM1* | Residues 1-311 of *dipA* fused to TEM1 under the control of the *omp26* promoter region | This study |
| pFNLTP6*omp26*-*dipACC(AIL^3^D)-TEM1* | *dipA* coiled-coil domain mutant A317D, I324D, L331D fused to TEM1 under the control of the *omp26* promoter region | This study |
| pFNLTP6*omp26*-*dipACC(LAL^3^D)-TEM1* | *dipA* coiled-coil domain mutant L414D, A317D, L321D fused to TEM1 under the control of the *omp26* promoter region | This study |

*References*

1. Maier TM, Havig A, Casey M, Nano FE, Frank DW, et al. (2004) Construction and characterization of a highly efficient Francisella shuttle plasmid. Appl Environ Microbiol 70: 7511-7519.

2. Charpentier X, Oswald E (2004) Identification of the secretion and translocation domain of the enteropathogenic and enterohemorrhagic Escherichia coli effector Cif, using TEM-1 beta-lactamase as a new fluorescence-based reporter. J Bacteriol 186: 5486-5495.

3. Wehrly TD, Chong A, Virtaneva K, Sturdevant DE, Child R, et al. (2009) Intracellular biology and virulence determinants of Francisella tularensis revealed by transcriptional profiling inside macrophages. Cell Microbiol 11: 1128-1150.
