# Supplementary material for: Structure-Function Analysis of DipA, a Francisella tularensis Virulence Factor Required for Intracellular Replication
Source: PLoS One. 2013 Jun 26;8(6):e67965. doi: 10.1371/journal.pone.0067965 (PMC3694160; doi:10.1371/journal.pone.0067965)
Supplement: Table S2 — (DOCX) [file pone.0067965.s004.docx]

Table S2. Primers used in this study.

| **Primer** | **Sequence 5' - 3'** |
| --- | --- |
| **pFNLTP6*omp26* construction** | |
| JC801 | CTGGTACCTTTGGGTTGTCACTCATCG |
| JC823 | TGTCATATGTATTTTATACAAAAAGTCTTTG |
| **pFNLTP6*dipA-HA* construction** | |
| JC825 | CGTGCTAGCTGTGACGCTTGATGTTTTTG |
| JC826 | CGACTCGAGCTAAGCATAATCTGGAACATCATA |
| JC828 | ATCTGGAACATCATATGGATATTTAGAAGTCACCGCATTTTG |
| **pFNLTP6*omp26*-*iglA*-*HA* construction** | |
| JC981 | CCGGGCGGCCGCTAGCAGCTGTATAAACATTGTGTTATTGGCG |
| JC982 | ATCTGGAACATCATATGGATACTTACCATCTACTTGTTG |
| JC955 | CTAGTGGATCCTCGAGCTAAGCATAATCTGGAACATCATA |
| **pFNLTP6*omp26*-*iglI*-*HA* construction** | |
| JC941 | AATTCCCGGGCGGCCGCGAGGAGATTTATATGAGTCAG |
| JC955 | CTAGTGGATCCTCGAGCTAAGCATAATCTGGAACATCATA |
| TW52 | ATCTGGAACATCATATGGATATATGTCAAAAAGATCTTCAAAATAGTC |
| **pFNLTP6*dipAΔSel1ab-HA* construction** | |
| JC943 | GGTACCATATGAATTCGAGTTATGAGTTTGACAAA |
| JC955 | CTAGTGGATCCTCGAGCTAAGCATAATCTGGAACATCATA |
| JC956 | ATACGATCTAGCACCTTCTGGATCTT |
| JC957 | GGTGCTAGATCGTATCTAGATGCACGTAATGCTTAC |
| **pFNLTP6*dipAΔSel1cd-HA* construction** | |
| JC943 | GGTACCATATGAATTCGAGTTATGAGTTTGACAAA |
| JC955 | CTAGTGGATCCTCGAGCTAAGCATAATCTGGAACATCATA |
| JC958 | ACAAATGCTGGGGCTATACAGTATGGATAGT |
| JC959 | GCCCCAGCATTTGTTGTAA |
| **pFNLTP6*dipAΔCC-HA* construction** | |
| JC955 | CTAGTGGATCCTCGAGCTAAGCATAATCTGGAACATCATA |
| JC943 | GGTACCATATGAATTCGAGTTATGAGTTTGACAAA |
| JC960 | TTTTGTTGCCAGCTAGAAATATCTGGCATACAATACTC |
| JC961 | AGCTGGCAACAAAATGCGGTGACTT |
| **pFNLTP6*dipACC(ALL^3^D)-HA* construction** | |
| JC943 | GGTACCATATGAATTCGAGTTATGAGTTTGACAAA |
| JC955 | CTAGTGGATCCTCGAGCTAAGCATAATCTGGAACATCATA |
| TW143 | TGATAATTTATCTTTAGCAAGATTTCGCTGATCTTGCTTCAGACCATTATCAG |
| TW144 | GCTAAAGATAAATTATCAAAGCAAGAGGATGAATTAGCTACCAAAGAGCAGCAAAGTTTGAG |
| **pFNLTP6*dipACC(LAL^3^D)-HA* construction** | |
| JC943 | GGTACCATATGAATTCGAGTTATGAGTTTGACAAA |
| JC955 | CTAGTGGATCCTCGAGCTAAGCATAATCTGGAACATCATA |
| TW145 | GCATCATTTCGCTGATCTTGCTTATCACCATTATCAGAAATATCTGGCAT |
| TW146 | GGTGATAAGCAAGATCAGCGAAATGATGCTAAAATTAAATTATCAAAGCAAGAG |
| **pFNLTP6*omp26*-*flpA*-*HA* construction** | |
| JC824 | CCAGCTAGCCTTTTTAGCAGCATTACGCA |
| JC826 | CGACTCGAGCTAAGCATAATCTGGAACATCATA |
| JC827 | ATCTGGAACATCATATGGATATTTATTGTCACTTGCTAGAGT |
| **pFNLTP6*omp26*-*fopA-HA* construction** | |
| JC955 | CTAGTGGATCCTCGAGCTAAGCATAATCTGGAACATCATA |
| TW210 | ATTCCCGGGCGGCCGCGAATAGTTAAAAAGTATTT |
| TW211 | GTTAGCTTCTTTAAGTGGAGCTGATA |
| TW212 | CTTAAAGAAGCTAACTATCCATATGATGTTCCAG |
| **pFNLTP6*omp26*-*dipA-TEM1, pFNLTP6omp26*-*dipAΔSel1ab-TEM1,*** | |
| **pFNLTP6*omp26*-*dipAΔSel1cd*-TEM1, pFNLTP6*omp26*-*dipACC(ALL3D)-TEM1*** | |
| **and pFNLTP6*omp26*-*dipACC(LAL3D)-TEM*1 construction** | |
| JC825 | CGTGCTAGCTGTGACGCTTGATGTTTTTG |
| JC829 | GTGAAAGTAAAAGATGCTGAAGATCAG |
| JC830 | CCTCTCGAGTCACCAATGCTTAATCAGTGAGGC |
| JC832 | CAGCATCTTTTACTTTCACTTTAGAAGTCACCGCATTTTG |
| **pFNLTP6*omp26*-*dipAΔCC-TEM1* construction** | |
| JC829 | GTGAAAGTAAAAGATGCTGAAGATCAG |
| JC832 | CAGCATCTTTTACTTTCACTTTAGAAGTCACCGCATTTTG |
| JC940 | CTAGTGGATCCTCGAGTCACCAATGCTTAATCAGTGAGGC |
| TW136 | CCGGGCGGCCGCTAGCTGTGACGCTTGATGTTTTTG |
| **pFNLTP6*omp26*-*iglA*-*TEM1* construction** | |
| JC829 | GTGAAAGTAAAAGATGCTGAAGATCAG |
| JC940 | CTAGTGGATCCTCGAGTCACCAATGCTTAATCAGTGAGGC |
| JC979 | CAGCATCTTTTACTTTCACCTTACCATCTACTTGTTG |
| JC981 | GGTACCATATGAATTCAGCTGTATAAACATTGTGTTATTGGCG |
| **pFNLTP6*omp26*-*iglI*-*TEM1* construction** | |
| JC829 pCX340 | GTGAAAGTAAAAGATGCTGAAGATCAG |
| JC830 | CCTCTCGAGTCACCAATGCTTAATCAGTGAGGC |
| JC866 | CAGCATCTTTTACTTTCACTATGTCAAAAAGATCTTCAAAATAGTC |
| JC891 | CCAGCGGCCGCGAGGAGATTTATATGAGTCAG |
| ***fopA* deletion and chromosomal detection** | |
| TW202 | ATCCATACAGTCGACCGATAGGTAAGCAAATCGTC |
| TW203 | GTTAGCTTCTTTAAGTGGAGCCATCAAAAACTCCTTTAAATAC |
| TW204 | GCTCCACTTAAAGAAGCTAACTAATATCAATTT |
| TW205 | GAGACCGGCAGATCTCTAATCGCTTGTTGAGTGTCT |
| TW206 | GATTATGAATTTCAGATGTCAAGTAT |
| TW207 | AACATAGTTTGTTGTAATAACGT |
| TW208 | CGAATCAGCACGTTGTGGTTGT |
| TW209 | GCTAATAACTCAAATTCATAGCTTGTG |
| **FTT1407c deletion and chromosomal detection** | |
| TW151 | CGGTACCCGGGGATCCCACAGGTTTGGTTTGATAG |
| TW152 | TTTGACTTCCTTCTGCtGACATCTTTTGCCTCTGT |
| TW153 | GCAGAAGGAAGTCAAAAATGA |
| TW154 | TATCCATACAGTCGACCTATACCTGCTACTTCGCC |
| TW155 | TACTGAGAGTGTTCTTGAC |
| TW156 | TCTCTGCACTAACCATTTC |
| TW157 | TGAGGATTGGGAAGATAAGT |
| TW158 | ATCTATAACGATAGCATGGC |
| ***fbaB* deletion and chromosomal detection** | |
| RC407 | CGGTACCCGGGGATCCGCAATCAGGTAGTTTAGGAT |
| RC408 | TGGATCTAACTCACCAACTAAAGCCATTATTATCTCC |
| RC409 | GGTGAGTTAGATCCAATTGTTAAATAATAA |
| RC410 | TATCCATACAGTCGACGAAATAACTCTCCAGCAATAC |
| RC411 | GAGGGTGGTAATACTTCGC |
| RC412 | CAAAAAGCAGGAGGAGGAGAG |
| RC413 | GGCATTAAAGACCAAGTTTC |
| RC414 | GGTCTCCTGAAGTTATCCT |
